# The Endophytic Strain *Trichoderma asperellum* 6S-2: An Efficient Biocontrol Agent against Apple Replant Disease in China and a Potential Plant-Growth-Promoting Fungus

**DOI:** 10.3390/jof7121050

**Published:** 2021-12-08

**Authors:** Haiyan Wang, Rong Zhang, Yanan Duan, Weitao Jiang, Xuesen Chen, Xiang Shen, Chengmiao Yin, Zhiquan Mao

**Affiliations:** State Key Laboratory of Crop Biology, College of Horticultural Science and Engineering, Shandong Agricultural University, Tai’an 271018, China; 2020010070@sdau.edu.cn (H.W.); 2019110243@sdau.edu.cn (R.Z.); 2019010063@sdau.edu.cn (Y.D.); 2018110228@sdau.edu.cn (W.J.); xschen@sdau.edu.cn (X.C.); shenx@sdau.edu.cn (X.S.)

**Keywords:** ARD, *T. asperellum* 6S-2, endophytic, biocontrol, plant-growth-promoting, spore fertilizer

## Abstract

A study was conducted for endophytic antagonistic fungi obtained from the roots of healthy apple trees growing in nine replanted orchards in Shandong Province, China. The fungi were assessed for their ability to inhibit *Fusarium proliferatum* f. sp. *malus domestica* MR5, a fungal strain associated with apple replant disease (ARD). An effective endophyte, designated as strain 6S-2, was isolated and identified as *Trichoderma asperellum*. Strain 6S-2 demonstrated protease, amylase, cellulase, and laccase activities, which are important for the parasitic and antagonistic functions of pathogenic fungi. The inhibition rate of 6S-2 against *Fusarium proliferatum* f. sp. *malus domestica* MR5 was 52.41%. Strain 6S-2 also secreted iron carriers, auxin, ammonia and was able to solubilize phosphorus. Its fermentation extract and volatile substances inhibited the growth of MR5, causing its hyphae to twist, shrink, swell, and rupture. The antifungal activity of the 6S-2 fermentation extract increased with increasing concentrations. It promoted the production and elongation of *Arabidopsis thaliana* lateral roots, and the strongest effects were seen at a concentration of 50 mg/mL. A GC-MS analysis of the 6S-2 fermentation extract and volatile substances showed that they comprised mainly alkanes, alcohols, and furanones, as well as the specific volatile substance 6-PP. The application of 6S-2 spore suspension to replanted apple orchard soils reduced plant oxidative damage and promoted plant growth in a pot experiment. Therefore, the endophytic strain *T. asperellum* 6S-2 has the potential to serve as an effective biocontrol fungus for the prevention of ARD in China, and appears to promote plant growth.

## 1. Introduction

Apple replant disease (ARD) is a complex disease caused by multiple factors [1,2]. It is generally believed that an imbalance in a soil microbial community structure is the main cause of ARD [1,3], although the pathogenic fungi that cause ARD in different regions are not the same [4,5,6]. Previous research has shown that *Fusarium* is the main pathogen that causes ARD in the Bohai Bay area [7]. The specialized *Fusarium proliferatum* f. sp. Malus domestica MR5 (MW600437.1), which is associated with ARD, has recently been screened and identified (in review); it is highly pathogenic to apple roots. Traditional chemical fumigation and pesticide application are highly effective in preventing and controlling ARD, but their use has harmful effects on the environment [1,8]. At present, *Trichoderma*, *Bacillus*, *Pseudomonas*, and *Streptomyces* are widely used as biological control agents [9,10,11], in line with the concept of “double reduction” of fertilizers and pesticides. Compared to rhizosphere soil strains, endophytes can more effectively colonize plants and better adapt to environmental changes, making them more effective in disease suppression and growth promotion [12]. Volatile organic compounds (VOCs) that are produced by endophytic fungi have been used to control plant disease and promote plant growth [13,14,15]. For example, endophytic fungi, isolated from *Pelargonium graveolens*, *Melia azedarach*, *Chenopodium album*, and *Malva parviflora*, are rich sources of secondary metabolites for use in bio-control [13]. Almeida et al. found that endophytes were promising biological control agents because of their antagonistic activity toward the mycelial growth of grapevine trunk diseases (GTD)-associated fungi [16]. Some endophytic fungi can also promote plant growth by increasing plant access to nutrients (nitrogen, phosphorus, potassium, zinc, and iron), producing phytohormones, and increasing plant stress tolerance [14]. Poveda reported that microbial VOCs (MVOCs) were highly beneficial to plants and could be used in agriculture [15]; MVOCs were found to modulate plant and microbial growth, induce plant systemic resistance, and affect insects, nematodes, and other organisms [17].

*Trichoderma* is the most commonly used genus of beneficial microorganisms in agricultural production, and has shown strong biocontrol effects on plant diseases. Members of the genus have been shown to promote crop productivity [18], increase abiotic stress tolerance [19], and control nematodes [20], insects [21], and fungi [22]. Previous studies have shown that *Trichoderma* species can colonize the rhizosphere of *Brassicaceae* plants and promote crop productivity [19]. The application of both arbuscular mycorrhizal fungi (AMF) and *Trichoderma harzianum* significantly improved the productivity of Brassicaceae species [19]. *Trichoderma harzianum* and *Trichoderma atroviride* are commonly used as biocontrol agents [9]. *Trichoderma* inhibit a variety of plant diseases through competition, parasitism, the secretion of secondary metabolites, production of volatile substances, and induction of plant resistance; they also have specific growth-promoting functions [23,24,25,26,27,28]. Some *Trichoderma* are endophytic species that coexist in mutual benefit with plants [29]. The volatile substances (alcohols, ketones, alkanes, furans, pyrones, terpenes, etc.) produced by *Trichoderma* can diffuse through air spaces in the soil or directly induce the plant immune response to inhibit the growth and invasion of pathogenic fungi [30,31]. As a specific volatile substance of *Trichoderma*, 6-PP has shown strong antifungal activity [32]. In addition, the fermentation extract and volatile gases of *T. asperellum* have shown biocontrol activities and growth-promoting capabilities [33,34]. The use of *Trichoderma* and other filamentous fungi as biological control agents is a promising and durable biocontrol strategy against plant-parasitic nematodes in agriculture [20]. *Trichoderma* can control insect pests directly through parasitism and the production of insecticidal secondary metabolites, antifeedant compounds, and repellent metabolites [21]. Therefore, the use of *Trichoderma* in agriculture is effective not only against plant pathogens but also against insect pests, representing a potential future strategy for the development of sustainable agriculture [21,22].

*T. asperellum* has presented the potential for use in sustainable agriculture, but its specific biocontrol effects on the specialized *Fusarium* that causes ARD have not been reported previously. Therefore, we examined the phenomenon of “disease-suppressing soil” that often occurs in replanted orchards, with the specific objectives of (1) screening beneficial antagonistic endophytic strains from healthy and vigorous apple roots in replanted orchards, (2) identifying and characterizing the most effective endophytic strain, and (3) investigating its antifungal and plant-growth-promoting activities.

## 2. Materials and Methods

### 2.1. Experimental Design

#### 2.1.1. Field Soil Samples for Screening of Endophytic Fungal Strains

The study was performed in nine replanted apple orchards in the Yantai and Yiyuan regions that had been established for 15–25 years and replanted for 4–5 years. The orchards contained both healthy and vigorous trees and weak and dead trees. Root systems from healthy and vigorous trees were used to screen for endophytic antagonistic fungi, and rhizosphere soil samples from healthy and weak/dead trees (controls) were used to compare microbial community structures. Root samples were collected from healthy trees, and rhizosphere soil samples were collected from healthy and unhealthy trees, with five samples per tree and three trees from each of the nine orchards. Roots collected from each of the sampled trees were mixed, placed into a sterile plastic bag, and stored in a refrigerator at 4 °C. Endophytic fungi were screened from the roots within 36 h. The collected rhizosphere soil was passed through a 12-mesh sieve, mixed, and divided into three parts. The first part was quickly frozen in liquid nitrogen, then stored in a refrigerator at −80 °C for soil DNA extraction and T-RFLP analysis. The second part was placed in a refrigerator at 4 °C; it was used for the screening and separation of soil fungi and the determination of soil texture. The third part was naturally air-dried and stored at room temperature for the determination of the soil’s basic physical and chemical properties (hydrolyzable nitrogen, available phosphorus, available potassium, organic matter, and pH). The basic physical and chemical properties of the nine orchards soils are presented in Supplementary Table S1.

#### 2.1.2. Field Soil Samples for Pot Experiments

Experimental soil was taken from a 35-year-old apple orchard in Manzhuang, Daiyue District, Tai’an City, Shandong Province in China. The soil texture was brown loam. Soil nutrient contents included 3.22 mg·kg^−1^ ammonium nitrogen, 6.18 mg·kg^−1^ nitrate nitrogen, 45.76 mg·kg^−1^ available phosphorus, 57.65 mg·kg^−1^ available potassium, and 8.10 g·kg^−1^ organic matter. The soil was passed through a 10-mesh sieve and mixed well for later use.

The first greenhouse experiment was performed in April 2021. Fifty black plastic pots (7.0 cm × 5.0 cm × 8.5 cm) were filled with old apple-orchard soil and planted with healthy T337 (apple rootstock) tissue culture seedlings, with 4–5 leaves. After 3 days of acclimation, 25 pots were irrigated with 1% 5 × 10^8^ CFU/g 6S-2 spore suspension, and the other 25 pots were irrigated with an equal volume of sterile water to serve as controls. After 15 days of growth under the same conditions, leaves and roots were harvested for nitro blue tetrazolium (NBT) and 3,3′-diaminobenzidine (DAB) staining and microscopy observation (Olympus, BX53, Pooher Laboratory Technology, Shanghai, China). After obtaining the results of the first greenhouse experiment, an outdoor pot experiment was performed in May 2021. Thirty white plastic pots (150 cm × 90 cm × 115 cm) were filled with old apple orchard soil and planted with *Malus hupehensis* Rehd. seedlings with 5–6 true leaves and no pests or diseases. The pots were treated with spore suspension or sterile water as described above. After a period of growth, photosynthetic parameters were measured on 25 July 2021. Destructive samplings were then used to measure plant biomass and root protective enzyme activities in the different treatments, and changes in the numbers of soil microorganisms were also determined.

### 2.2. Measurement Index

#### 2.2.1. Soil-Related Indicators

##### Soil DNA Extraction and Terminal-Restriction Fragment Length Polymorphism (T-RFLP) Analysis

The total DNA was extracted from 0.5 g of soil using an E.Z.N.A. soil DNA kit (Omega Bio-tek, Norcross, GA, USA), according to the method of Wang et al. [7]. A T-RFLP analysis was performed to compare fungal community structure between the healthy and unhealthy rhizosphere soils. The primers used to amplify the fungal ITS region were ITS1-F-FAM (5′-CTTGGTCATTTAGAG GAAGTAA-3′) and ITS4 (5′-TCCTCCGCTTATTGATAGC-3′). The ITS amplification reaction system consisted of 12.5 μL 2× Taq Master Mix, 1 μL of the DNA template, 1.5 μL each of ITS1-F and ITS4 (10 μM), and sterile water, to 25 μL. The PCR reaction conditions were 94 °C pre-denaturation for 3 min; 34 cycles of 94 °C denaturation for 60 s, 51 °C annealing for 60 s, and 72 °C extension for 60 s; and a final 72 °C extension for 10 min. Five microliters of the ITS-PCR amplification product were separated onto a 2% agarose gel, following the instructions of the PCR product purification kit (FastPure Gel DNA Extraction Mini Kit DC301-01, Vazyme, Nanjing, China) and stored at −20 °C for later use. Next, the purified PCR product was digested with the restriction enzyme Hha I. The 30-μL reaction system contained 10 μL of the purified ITS-PCR product, 2 μL HhaI (10 U/μL), 2 μL 10× buffer, and double-distilled water, to 30 μL. The reaction was placed in a 37 °C water bath and incubated for 4 h. After digestion, the water bath temperature was raised to 65 °C for 20 min to terminate the reaction. The digested products were sent to Shanghai Shenggong Biotechnology Co., Ltd. (Shanghai, China) for sequencing [35]. The T-RFLP profiles were analyzed, and the fragments were misprinted with a 1-nt margin using the AFLP tool in PAST v. 3.2.5 fragment analysis software [36]. A principal component analysis (PCA) of healthy and unhealthy soil samples from the nine sites was performed using PAST.

##### Soil Culturable Microorganisms and Real-Time Fluorescence Quantification of Four Fusarium Species

The culturable bacteria, fungi, and actinomycetes in the soil were counted using the dilution plate method [37] with 10 g of fresh soil stored at 4 °C for no more than 24 h. The gene copy numbers of *F. oxysporum*, *F. proliferatum*, *F. solani* and *F. moniliforme* in the soil were determined using a CFX96TM Thermal Cycler (Bio-Rad, Beijing, China) [7].

#### 2.2.2. Isolation of Endophytic Fungi Antagonistic to MR5 and Verification by Re-Screening

##### Isolation of Endophytic Fungi from Healthy Apple Roots

Fungal endophytes were isolated from the roots of healthy apple trees immediately after collection. The isolation protocol was optimized as described in Su et al. [38] and Manias et al. [39]. The roots were washed under running tap water for 5 min to remove adhering soil particles. They were surface-sterilized by dipping in 75% ethanol for 2 min and rinsing three times with sterile water, then placed in a 5% sodium hypochlorite solution and shaking was performed for 5 min, followed by rinsing five times in sterile distilled water. After careful drying, the surface-sterilized roots were cut into 1 cm sections and transferred to the potato dextrose agar medium (PDA) with four per thousand penicillin streptomycin (100×) to isolate the endophytic fungi. Four roots were placed in each Petri plate, and the plates were maintained at 28 °C for one week. Sterile water from the final root rinse was also plated and incubated to confirm that the roots were completely disinfected.

After screening, separating, and disinfecting the roots, we smeared sterile water used for the last surface disinfection and washing onto PDA plates; no fungi were produced on these plates. Healthy apple-root tissues were cultured for 3–4 d after surface disinfection, and hyphae gradually became visible. The fungal tissue grew from the cut ends of the root samples. Sixty-one culturable fungi were isolated from healthy apple roots from the nine regions. After pure culturing the fungi, we performed morphological comparisons and obtained a total of 15 endophytic fungi.

##### Dual Culture Test with MR5

A plate confrontation test was used to assess the antifungal ability of the 15 endophytes against the specific *Fusarium proliferatum* f. sp. *malus domestica* MR5 that causes ARD in China. Tests were performed according to the method of Hewedy et al. [40]. The MR5 was isolated and preserved in the laboratory, and is currently under conditions of confidentiality. In a preliminary antagonism test, 6S-2 had the best antagonistic effect towards MR5, producing a 6 mm antagonistic circle. Therefore, the following experiments focused on the antagonistic endophytic fungi 6S-2.

##### Re-Isolation of Endophytes

We used the method of Wu [41] to optimize the procedure for re-isolation of 6S-2 and confirm its endogeneity. Using this method, 6S-2 was cultivated on a PDA medium at 28 °C for 6 days until the spores had grown; 10 mL of sterile water was then added to the center of the plate, and the spores were gently scraped with a coating rod to create a spore suspension. After filtering through four layers of sterilized lens-cleaning paper to remove excess hyphae, 1 μL of filtrate was added dropwise to the center of a hemocytometer, and spores were counted under a microscope. Finally, sterile water was used to dilute the spore solution to a concentration of 3 × 10^6^ CFU/mL. We selected 20 bottles of rooted T337 tissue culture seedlings of similar growth status. Ten bottles were placed on an ultra-clean workbench and soaked in the 6S-2 spore suspension for 2 h. The seedlings were then transplanted into containers with 3 g of high-temperature-sterilized substrate and watered with 5 mL of the 3 × 10^6^ CFU/mL suspension. The remaining ten bottles were soaked in sterile water for 2 h, transplanted, and watered with the same volume of sterile water. The seedlings were placed in a light incubator (25 °C, 12 h/12 h). Seven days later, the initial endophyte isolation method was used for screening, and PAS (Periodic Acid-Schiff) staining was performed [42].

#### 2.2.3. Strain Identification

##### Morphological Identification

Strain 6S-2 was inoculated into the PDA medium, Charpy medium, CM medium, CMD medium, and SNA medium, and its morphology was observed under an optical microscope (Olympus, BX53) and a scanning electron microscope (Hitachi, SU8100, RECO, Beijing, China). The morphology of the specific branching form of the conidiophores was recorded.

##### Molecular Biology Identification

DNA was extracted from 6S-2 using the Fungi Genomic DNA Extraction Kit (Solarbio Cat#D2300, Solarbio Life Sciences. Beijing, China). The ITS fragment was amplified with the primer pair ITS1 (5′-TCCGTAGGTGAACCTGCGG-3′) and ITS2 (5′-GCTGCGTTCTTCATCGATGC-3′) [43]. Thermal cycling was performed with the following parameters: a 1 min initial denaturation at 94 °C; 30 cycles of 1 min denaturation at 94 °C, 1 min primer annealing at 50 °C, and 90 s extension at 74 °C; and a final extension at 74 °C for 7 min. The Tef1 fragment was amplified with the primer pair tef1fw (5′-GTGAGCGTGGTATCACCATCG-3′) and tef1rev (5′-GCCATCCTTGGAGACCAGC-3′) using the following parameters: a 1 min initial denaturation at 94 °C; 30 cycles of 1 min at 94 °C, 1 min at 59 °C, and 50 s at 74 °C; and a final extension at 74 °C for 7 min [44]. The kit was used to recover and purify the PCR amplification products, which were sent to Shanghai Shenggong Sequencing Co., Ltd. for sequencing.

The sequencing results were analyzed with SnapGene 4.2.4, and the sequences were compared with those in GenBank (http://www.ncbi.nlm.nih.gov/BLAST, accessed on 1 August 2021) using NCBI BLAST (version 2.2.12). The ITS and Tef1-α sequences of the known strains were compared and analyzed for homology. A phylogenetic tree of the ITS and Tef1-α sequences was constructed in MEGA 7.0 software using level 3, the maximum likelihood method, and 1000 bootstrap replicates. ITOL software (https://itol.embl.de, accessed on 7 August 2021) was then used for tree optimization.

#### 2.2.4. Media

The media used included PDA, Charpy, CMD, SNA, phosphate (NBRIP) broth, amylase, chromeazurol S (CAS), laccase, cellulase culture, protease culture, ammonia production, and half-strength Murashige and Skoog (MS) media. The details of specific media formulas are presented in Supplementary Table S2.

#### 2.2.5. Preparation of 6S-2 Liquid Fermentation Extract

The extraction conditions for 6S-2 fermentation materials were further optimized based on the method of Yang et al. [45]. The 6S-2 that was stored in the slope of a test tube was activated on a PDA plate and then re-inoculated into a new PDA culture. After 7 d, once spores had been produced, the culture was rinsed with sterile water to produce a 10^6^/mL spore suspension, then added to the PDB medium at a rate of 1%. It was then cultured at 160 rpm and 28 °C for 8 d. After filtering through a layer of gauze, the culture was centrifuged at 12,000 rpm at room temperature for 5 min, and the supernatant was collected and extracted with ethyl acetate in a 1:1 ratio. The excess water in the extract was absorbed with anhydrous calcium sulfate before being concentrated to a dry powder using a rotary evaporator (36 °C, 80 hPa). The resulting powder (100 mg), derived from the 6S-2 liquid fermentation extract, was re-dissolved in 10 mL methanol to produce 10 mg/mL mother liquor, centrifuged at 10,000 rpm, filtered through a 0.22-μm Nylon66 membrane to remove impurities, and stored in a refrigerator at 4 °C for later use. In cases for which longer term storage was required, the mother liquor was stored in a freezer at −80 °C. It was then added to 100 mL of the PDB solution and diluted to various concentrations for use.

#### 2.2.6. Production of 6S-2 Spore Powder

The 6S-2 that was stored in the tube was inoculated onto a PDA medium and grown for 5 days until spores were visible; then, the 6S-2 spore powder was expanded using the shallow-plate-fermentation method [46]. Four hundred grams of sterile medium (wheat bran and corn flour in a 4:1 volume ratio with 45% sterile water added) was placed in a shallow plate (30 cm × 20 cm × 5 cm), 2% solid 6S-2 inoculum was added, and the plate was covered with sterilized double gauze. The shallow plate was incubated at 28 °C and turned once every 2 d; a humidifier was used to maintain humidity. After 12 days of fermentation, the culture was air-dried, pulverized, and sieved to obtain spore powder of *T. asperellum* 6S-2. The specific fermentation process is shown in Supplementary Figure S1. The concentration of the resulting spore powder was 1.05 × 10^9^ CFU/g. It was stored in a sealed bag and diluted to 5 × 10^8^ CFU/g with sterile water before use.

#### 2.2.7. Antifungal Tests

##### Plate Confrontation Test with Multiple Pathogenic Fungi

A plate confrontation test was used to assess the antifungal ability of endophytes against multiple pathogenic fungi. Endophytes shared by the healthy apple roots from nine regions were tested with 12 types of harmful pathogenic fungi. This method was based on the study of Hewedy et al. [40]. Seven days later, the inhibition rates of 6S-2 towards the 12 pathogens were calculated as follows: Inhibition rate = (radius of the control pathogen − radius of the pathogen co-cultured with 6S-2)/radius of the control pathogen × 100%. The control MR5 hyphae and the hyphae cultured with 6S-2 were then stained with PI (propidium iodide) [47], and hyphal luminescence was observed under green fluorescence (450–490 nm), and hyphae at the junction of 6S-2 and MR5 were picked. Scanning electron microscopy was also performed.

##### Cellophane Culture Antifungal Test

Sterile cellophane film was spread onto plates containing PDA medium. A fungal cake of 6S-2 (activated for two generations) was placed in the center of each plate in the treatment group, and PDA agar blocks of the same size were placed in the center of the control plates. After 7 d of culture, the cellophane was removed, and an MR5 fungal cake (activated for two generations) was placed on the surface of each plate. When the MR5 hyphae had grown to cover the control plates, the radii of the colony in the treated plates were measured, and the hyphae were observed under a microscope [48].

##### Oxford Cup Test

The Oxford cup antifungal experiment was performed using the method described in Ahsan et al. [49]. A layer of 2% water-agar medium was poured onto the bottom of a Petri plate, two Oxford cups were placed 1 cm away from the edge, and 1% volume MR5 spore solution (10^5^ CFU/mL) was mixed with a PDA medium (cooled to 50 °C), and poured over the surface of the water agar. After solidification, the Oxford cups were removed, and 20 μL of the 6S-2 fermentation extract was added to the left side. An equal volume of methanol and sterile water was added to the right side as a control.

##### Antifungal Experiment with Different Concentrations of Liquid Fermentation Extract

The 6S-2 mother liquor was mixed with a PDA medium cooled to 50 °C in different proportions, then diluted to 50 mg/L, 100 mg/L, 150 mg/L, and 200 mg/L. Blank controls without 6S-2 were also prepared. After the culture medium had solidified, a second-generation activated MR5 fungal cake was placed in the center of the medium. After the MR5 in the control medium had grown to cover the entire Petri plate, the mycelial growth radii of the different treatments were measured, and the inhibition rates were calculated [50].

##### Antifungal Experiment with 6S-2 Volatile Substances

A two-part plate was used for the antifungal test of volatile substances [51]. The MR5 was inoculated on the right side of a two-part plate, 6S-2 was inoculated on the left side, and the plate was sealed with liquid paraffin. For the control group, MR5 was inoculated on the right side only. The plates were wrapped with parafilm and incubated at 28 °C for 5 d, then observed and photographed. Afterwards, an improved volatile antibacterial test was performed, using the sandwich plate technique [51]. The PDA medium was poured onto both the bottom and the lid of a Petri plate; MR5 was inoculated on the bottom, and 6S-2 was inoculated on the lid. For the control, a blank agar block was placed on the lid. Plates were sealed with liquid paraffin, wrapped with parafilm, incubated at 28 °C for 7 d, and then observed and photographed. In addition, the culture medium on the lid of the Petri plate was divided into two with a sterile scalpel. The culture medium on the left side was removed and replaced with activated charcoal, and the right side was inoculated with 6S-2. The bottom of the plate was inoculated with MR5, sealed with liquid paraffin, wrapped with parafilm, incubated at 28 °C for 7 d, and then observed and photographed. The MR5 hyphae were picked for observation under a light microscope and a scanning electron microscope, as described for the liquid fermentation extract experiment.

##### Spore Germination Test

Based on the method of Hu et al. [52], 30-μL aliquots of MR5 suspension containing 10^5^ CFU/mL was combined with equal volumes of 6S-2 fermentation extracts to serve as the treatment group. The control group contained equal volumes of methanol and MR5. After incubating in a 28 °C incubator on a gravure slide for 36 h, spore germination was observed under a microscope.

#### 2.2.8. Microscopy and Electron Microscopy Observations

For hyphal observation, a drop of lactophenol cotton blue dye solution was placed on a glass slide. Hyphae from the control and treatment groups were picked and smeared evenly in the dye solution, then covered with a cover glass. Excess dye solution was absorbed with absorbent paper, and the glass slides were baked and fixed with an alcohol burner, then placed under the microscope for hyphal observation. Additional hyphae were picked and placed in an electron microscopy fixing solution for 12 h, then sent to Wuhan Servicebio Technology Co., Ltd. (Wuhan, China) for SEM sample observation [53].

#### 2.2.9. Analysis of Liquid Fermentation Extract and Volatile Substance

Gas chromatography–mass spectroscopy (GC-MS) was used to determine the composition of the liquid fermentation extract as described in Wu et al. [54]. Headspace solid phase micro-extraction (HS-SPME) was used to collect 6S-2 volatile metabolites and to perform the component analysis as described in Kottb et al. [55]. After 6S-2 had grown on PDA medium for 6 d, a key was used to gently scrape the spores from the surface of the culture, avoiding any scratching of the culture medium. This step was performed on an ultra-clean workbench. The spores were placed in the lower third of a 20 mL headspace sample bottle (Welch, Concord, MA, USA) with a Teflon pad cover. The cap was quickly closed and sealed, and the bottle was placed in a 28 °C incubator for 48 h. Based on the principle of similar compatibility, the blue (SPME) 65micron polydimethylsiloxane (PDMS) extraction head (Supelco, Sigma-aldrich, St. Louis, MO, USA) was selected. It was aged at 250 °C for 30 min before use until there were no impurity peaks. When collecting volatile gases, the headspace bottle was first fixed with an iron stand, then placed in a 40 °C water bath for 30 min. The needle tube was pierced into the headspace bottle and fixed; the handle rod was pushed to extend the fiber head in the extraction head. The needle tube was placed in the upper portion of the headspace without touching the sample, and collection continued for 40 min. After collection, the extraction head was removed, and the needle tube was pushed out of the sample bottle and used for sample injection. The specific procedures are detailed in Supplementary Table S3.

The experimental results were compared with spectra in the NIST 17 database, and peak-area normalization was used to express the relative content of each metabolite as the ratio of the peak area of each volatile product to the total peak area. The triple quadrupole gas chromatograph-mass spectrometer (GCMS-TQ8040 NX) and peak-processing software were obtained from Shimadzu (Beijing, China). The retention time was used for qualitative identification, and the peak areas of external standards were used for quantification.

#### 2.2.10. Plant Related Indicators

##### *Arabidopsis thaliana* Disinfection

To prevent infection with microorganisms during the cultivation process, the method of Ren et al. [56] was used with some modifications to disinfect *Arabidopsis thaliana* seeds. *Arabidopsis thaliana* seeds were placed in a refrigerator at 4 °C for 3 d, then divided into sterile centrifuge tubes. They were rinsed twice with sterile water, placed in 75% alcohol for 3 min, rinsed three times with sterile water, placed in 40% sodium hypochlorite for 1 min, and finally rinsed five times with sterile water to complete the disinfection process.

##### Growth Promotion Experiment with Liquid Fermentation Extract

The effect of the 6S-2 liquid fermentation extract on *Arabidopsis thaliana* growth was assessed by the method described by Hernández et al. [57] to rapidly determine whether the extract had the potential to promote plant growth. Seeds were sown on ½ MS medium containing different concentrations of 6S-2 liquid fermentation extract in methanol (10, 50, 100, 150, and 200 mg/L). Equal amounts of methanol were used as controls, and blank controls were prepared without the addition of any substances. Five seeds were sown per plate. The plates were blown dry on a sterile workbench and placed in an illuminated culture box at 23 °C, with a 16 h light/8 h dark photoperiod. After 9 d of cultivation, the root length and lateral root length of the *Arabidopsis thaliana* seedlings were measured with a ruler, their fresh weights were obtained with an electronic balance, and the numbers of lateral roots were counted.

##### Growth Promotion Experiment with Volatile Substances

A two-part plate was used to rapidly detect whether the volatile substances of 6S-2 had the potential to promote *Arabidopsis thaliana* growth [58]. Half-strength MS medium was poured on the left side of a two-part plate, and a PDA medium was poured on the right side. Three sterilized seeds were placed on the ½ MS medium, and the 6S-2 spore solution (10^6^ CFU/mL) was evenly smeared on the right side. Sterile water was smeared as a blank control, and each treatment was replicated five times. The plates were sealed with liquid paraffin, wrapped with parafilm, cultured in a light incubator for 9 d, and observed. The root length and lateral root length were measured with a ruler, the fresh weight was obtained with an electronic balance, and the number of lateral roots were counted.

##### Nitro Blue Tetrazolium (NBT) Staining

The NBT histochemical staining method allows for a rapid and intuitive observation of reactive oxygen species accumulation in tissues and the qualitative observation of the degree of tissue-oxidative damage. With reference to the method of Javvaji et al. [59], nitro blue tetrazolium (200 mg) powder was added to PBS (100 mL), incubated at 37 °C for 30 min, and vortexed briefly to dissolve. Ten healthy leaves and roots from T337 seedlings of different treatments were soaked in a 0.2% NBT staining solution, then placed in a water bath at 37 °C for 2 h. Tissues were de-stained with 80% alcohol in water at 80 °C, rinsed with water, and photographed.

##### DAB (3,3′-Diaminobenzidine) Staining

The DAB chemical staining method allows for a rapid and intuitive observation of hydrogen peroxide accumulation in tissues, allowing for the rapid detection of sites at which peroxidase is active. The DAB color reagent kit (20×, DA1010, Solarbio) was used to stain healthy leaves and roots from T337 tissue culture seedlings of different treatments. Five milliliters of solution A and of solution B were mixed, diluted to 100 mL with 90 mL PBS, and stored in the dark. Leaves and roots were immersed in this solution, then placed in a water bath at 37 °C for 1 h. The tissues were de-stained with 80% alcohol in water at 80 °C, rinsed with water, and photographed [60].

##### *M. hupehensis* Seedling Growth and Biomass

Plant growth and biomass are simple and informative phenotypes for characterizing differences among treatments. We therefore measured the plant height and stem diameter of *M. hupehensis* seedlings with a meter ruler and vernier calipers, respectively. The dry and fresh weights of seedlings were measured using an electronic balance. Images of roots from *Malus* seedlings were obtained with a Scan Maker i800 plus scanner (Microtek. Shanghai, China), and various root system parameters were measured using an LA-S plant image analyzer (Hengmei Electronic Technology, Weifang, China).

##### Photosynthetic Characteristics of *M. hupehensis* Seedlings

The photosynthetic capacity of plants is closely related to plant growth. The level of photosynthesis directly affects the yield and quality of crops. The intercellular CO_2_ concentration (*C*_i_), net photosynthetic rate (*P*_n_), stomatal conductance (*G*_s_), and transpiration rate (*T*_r_) of *M. hupehensis* seedlings were measured using a CIRAS-3 portable photosynthesis instrument (PP System, UK, INCH, Beijing, China) at 10 a.m. on 25 July 2021 [61].

##### Root Protective Enzyme Activities and Malondialdehyde (MDA) Content of *M. hupehensis* Seedlings

Superoxide dismutase (SOD), peroxidase (POD), and catalase (CAT) activities are the core of the plant’s antioxidant system. SOD converts the superoxide radical into H_2_O_2_ and O_2_, and CAT and POD remove excess H_2_O_2_, thereby limiting plant oxidative damage. MDA is one of the major products of membrane lipid peroxidation, and the MDA content can therefore be used as an indirect measurement of membrane lipid peroxidation to assess plant stress resistance. Fresh white roots of *M. hupehensis* seedlings were rinsed and quickly frozen in liquid nitrogen to determine the activity of root protective enzymes. The method used to determine SOD activity was based on Sun et al. [62]. POD activity was measured using the guaiacol method as described by Omran [63]. CAT activity and MDA content were measured following the method of Singh et al. [64]. 

Each treatment was replicated three times, and three plants were measured per replicate.

## 3. Results

### 3.1. T-RFLP Analysis of Soil Fungi

The T-RFLP analysis indicated that there was a significant separation between the rhizosphere soil fungal community of healthy fruit trees and that of unhealthy fruit trees, indicating that their fungal community structures were different (Supplementary Figure S2). In the context of this experiment, it is important to characterize these differences before screening endophytes because endophytes are likely to derive from rhizosphere soil.

### 3.2. Isolation of Endophytic Fungi Antagonistic to MR5 and Verification by Re-Screening

After screening, separating, and disinfecting the roots, a total of 15 endophytic fungi were obtained. ITS sequencing and identification indicated that they belonged to *Trichoderma*, *Mucor*, *Mortierella*, *Fusarium*, *Aspergillus*, *Penicillium*, and *Chaetomium*.

**Figure 1:**
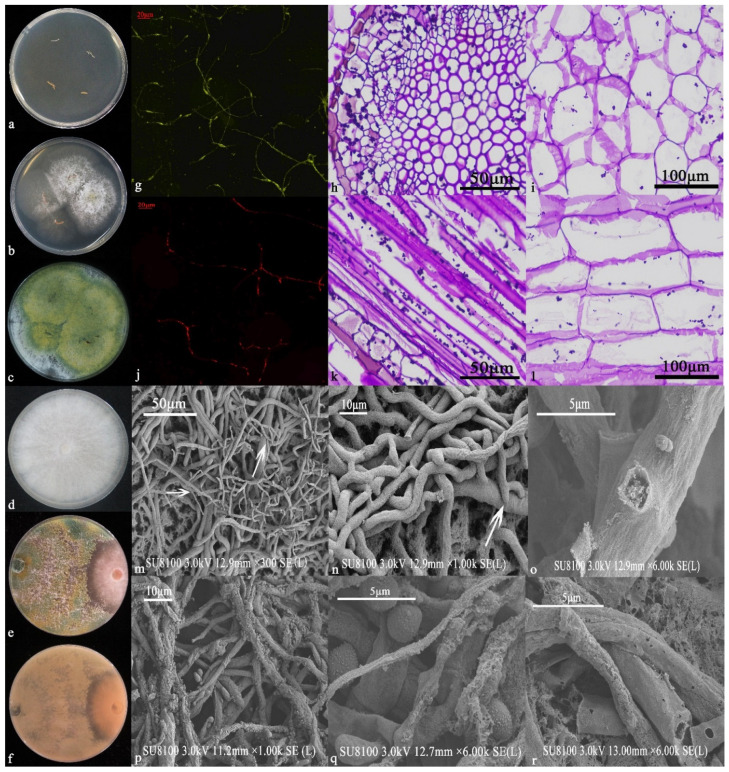
Screening re-isolation: control (**a**), after culture for 3 d (**b**), and after culture for 5 d (**c**). 6S-2 and MR5 antagonism test: MR5 control (**d**), front side of antagonism test (**e**), and reverse side of antagonism test (**f**). PI staining: MR5 control (**g**), and MR5 cultured against 6S-2 (**j**). PAS staining: cross section 100× (**h**), cross section 400× (**i**), longitudinal section 100× (**k**), and longitudinal section 400× (**l**). SEM observations of MR5 hyphae after co-cultivation with 6S-2 (**m**–**r**).

After inoculating 6S-2 onto T337 tissue culture seedlings, the seedlings showed no signs of disease. The 6S-2 inoculation produced consistent hyphal growth, whereas the control inoculation did not produce any fungi (Figure 1a). After 3 d of culture, white hairy hyphae formed around the roots of the T337 seedlings (Figure 1b), and after 5 d, yellow-green spores appeared on the hyphae (Figure 1c). A preliminary antagonism test revealed that 6S-2 had an antagonistic effect on MR5 (Figure 1d–f). Under 450–490 nm illumination, the MR5 control hyphae showed green fluorescence (Figure 1g), indicating that the hyphae were intact and not significantly damaged. By contrast, the hyphae co-cultured with 6S-2 showed red fluorescence (Figure 1j), indicating that the structure of the hyphae was damaged. After PAS staining, purple-red globular spores could be detected in the root cross sections (Figure 1h,i) and longitudinal sections (Figure 1k,l), indicating that 6S-2 had colonized the T337 tissue culture seedlings, further characterizing 6S-2 as a good endogenous antagonist. As a result, 6S-2 was used in subsequent tests. Scanning electron microscopy results showed that when MR5 and 6S-2 were cultured face-to-face, their hyphae began to cross. With time, the hyphae of 6S-2 grew close to those of MR5, producing a tangled structure (Figure 1m,n). Finally, the 6S-2 hyphae produced spores on the MR5 hyphae, forming perforations and causing the MR5 hyphae to rupture, release their contents, and finally shrink (Figure 1o,r). In addition, a large number of 6S-2 spores parasitized the hyphae of MR5 (Figure 1p,q).

### 3.3. Identification and Functional Analysis of 6S-2

#### 3.3.1. Morphological Identification and Functions

**Figure 2:**
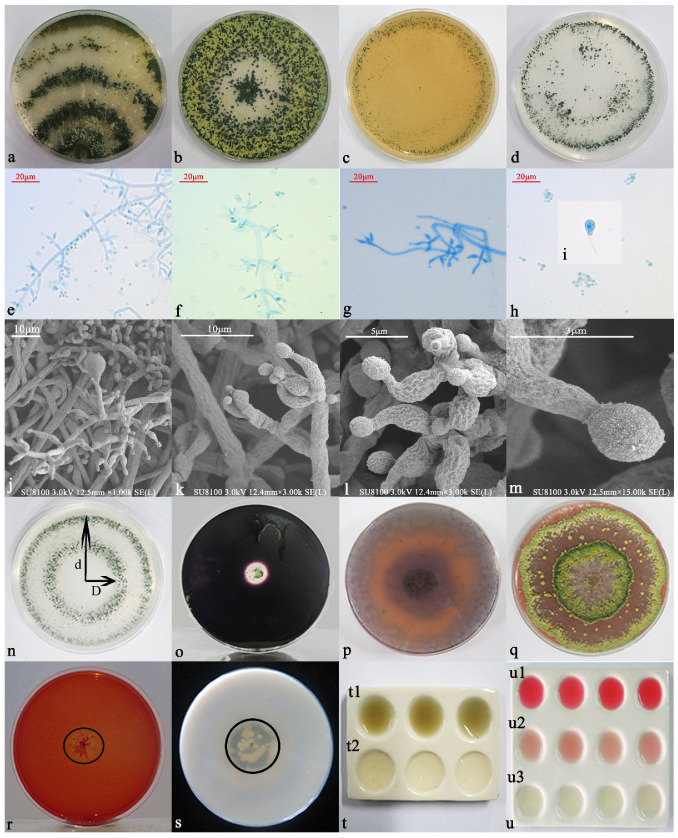
Morphological analysis of 6S-2 in PDA medium (**a**), Charpy medium (**b**), CMD medium (**c**), SNA medium (**d**). Sporangium morphology (**e**–**g**) and spore morphology (**h**) observed at 60× under an ordinary optical microscope. Scanning electron microscopy (SEM) images of spore stalk characteristic morphology (**j**–**m**) and spore morphology (**i**). Functional analysis of 6S-2: NBRIP medium (**n**), amylase medium (**o**), CAS medium (**p**), laccase medium (**q**), cellulase medium (**r**), protease medium (**s**), ammonia production medium (**t**), 6S-2 + Nesler reagent (**t1**), 6S-2 (**t2**), IAA production medium (**u**), IAA standard (**u1**), 6S-2 + Salkowski reagent (**u2**), and 6S-2 (**u3**).

When cultured on a PDA solid medium (Figure 2a), the initial hyphae were found to be white and wool-like, spreading to the edge, and the back of the colony was colorless. In the middle stage, the hyphae gradually became light green, and the spore ring formed on the early developing hyphae, gradually changing to yellow. The hyphae became green, and the aerial hyphae were more luxuriant, thicker, and had a cotton wool appearance. In the later period, the colony formed three white and green concentric rings with dense conidia attached to the rings, and the overall strain was dark green. Closer to the center, the color became darker and fewer aerial hyphae appeared. The plate can become overgrown in 5 d. When observed under an ordinary optical microscope at 60×, the hyphae were found to be slender and separable, and the main branches were dendritic and slender. Conidiophores are branched and produced in pustules, and secondary branches formed. The primary branches beneath the top of the main shaft often appear to be opposite one another, and the angle with the main axis was found to be nearly 90°. With increasing distance from the top, the length of the first branch also increased. The length of each pair of branches was almost equal, and the second branch that formed near the main axis was the longest. The second branch can produce a third branch, but we found that the third branch does not branch any further. The phial stems on the conidiophores were distributed symmetrically and were produced at the top of the primary, secondary, and tertiary branches. The phial stalks are short. At the tip, the phial stalk shrunk sharply and became thinner; in the image, it can be seen that it bends and swells in the middle, showing an ampoule shape with 3 or 4 groups of whorls at the top, arranged in a whirlpool pattern. After maturity, conidia were produced at the tip of the phial stem (Figure 2e–g). Phialides were found to be 5.24–9.75 µm long and 2.00–2.55 µm wide at the base. Conidia (2.35–3.95 × 2.08–3.43 µm) were green and spherical or ovoid, clustered or scattered, with fine thorns on the wall (Figure 2h–m). Chlamydospores appeared, and the Charpy medium was found to be conducive to sporulation. Yellow pustules formed on the plate at the initial stage; they gradually matured and became green in the later stage, finally forming a yellow-green ring (Figure 2b). In the CMD medium and SNA medium, the morphology was found to be essentially the same, whereby the hyphae are thin, and the colony is transparent and radiant. It was able to cover the whole medium in around 3 d. In the CMD medium, the blister-like structure was more aggregated and compact, whereas in the SNA medium, the blister-like structure was more dispersed, with a semi-circular or flaky shape (Figure 2c,d).

Inoculation and cultivation on different functional media indicated that 6S-2 had multiple functions. The ratio of the phosphate ring diameter (D) to the colony diameter (d) was 0.67, and 6S-2 therefore appears to be able to degrade insoluble phosphate and release soluble phosphate (Figure 2n). The 6S-2 had strong amylase and protease activities that play a role in degrading the lipid and protein components of the cell wall, thereby enhancing its growth competitiveness (Figure 2o,s). Furthermore, 6S-2 also showed a weak cellulose-degradation ability (Figure 2r). After incubation on PDA plates for 7 d, the colony was covered with CAS medium and incubated for 2 h, and the color of the covering medium changed from blue to orange, indicating that 6S-2 could produce hydroxy-type siderophores (Figure 2p). The color change of PDA medium with guaiacol indicated that 6S-2 possesses laccase activity (Figure 2g). In the process of growth, 6S-2 produced ammonia gas and IAA (Figure 2t,u).

#### 3.3.2. Molecular Biology Identification

The ITS sequence length of strain 6S-2 is 265 bp (GenBank accession number MZ841617), and its Tef1-α sequence length is 262 bp (GenBank accession number JQ040445). Known sequences of *T. asperellum* SZMC:24288 (GenBank ITS accession number MN516477.1, TEF1 accession number N520032.1) were up to 100% identical to those of 6S-2, and 6S-2 is morphologically consistent with the description of the model strain. We therefore concluded that 6S-2 is *T. asperellum*. See Supplementary Table S2 for details of the strains used for phylogenetic-tree construction.

### 3.4. Analysis of 6S-2 Antagonistic Activity

#### 3.4.1. Analysis of Antagonistic Activity by Plate Confrontation

**Figure 3:**
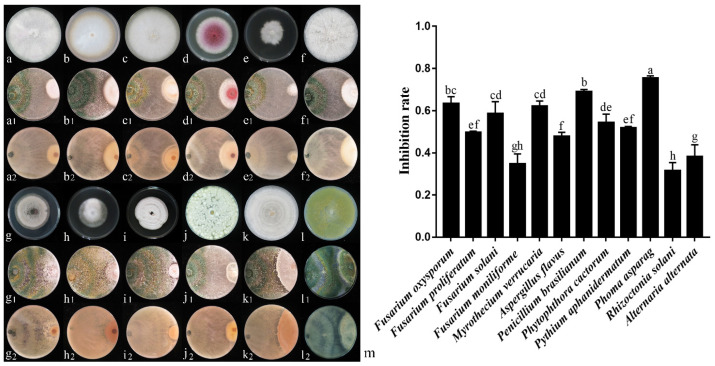
In vitro dual culture assay of 6S-2 against different pathogenic fungi on PDA medium at 7 d. In the confrontation culture, the left side was inoculated with 6S-2, and the right side was inoculated with a pathogenic fungus: *Fusarium oxysporum* (**a**), *Fusarium proliferatum* (**b**), *Fusarium solani* (**c**), *Fusarium moniliforme* (**d**), *Phytophthora cactorum* (**e**), *Pythium aphanidermatum* (**f**), *Phoma asparagi* (**g**), *Alternaria alternata* (**h**), *Myrothecium verrucaria* (**i**), *Penicillium brasilianum* (**j**), *Rhizoctonia solani* (**k**), and *Aspergillus flavus* (**l**). Bar chart of 6S-2 inhibition of the growth rates of different pathogenic fungi (**m**). (**a**–**l**) is the control figures of 12 kinds of pathogen fungi; (**a**_1_–**l**_1_) is the frontal of 6S-2 inoculated with 12 kinds of pathogenic fungi, and (**a**_2_–**l**_2_) is the negative of 6S-2 inoculated with 12 kinds of pathogenic fungi. Different lowercase letters in the same column indicate a significant difference at *p* < 0.05 level by Duncan’s new multiple range test.

In the study, 6S-2 was cultured with 12 harmful pathogens for 7 d and was able to inhibit *Fusarium oxysporum*, *Fusarium proliferatum*, *Fusarium solani*, *Fusarium moniliforme*, *Phytophthora cactorum*, *Pythium aphanidermatum*, *Phoma asparagi*, *Alternaria alternata*, *Myrothecium verrucaria*, *Penicillium brasilianum*, *Rhizoctonia solani*, and *Aspergillus flavus* to varying degrees. The 6S-2 inhibition rate of mycelial growth was 31.48–75.37% (Figure 3m), and it produced antagonistic transparent circles of different diameters. Some harmful strains showed parasitic 6S-2 spores on their hyphae, and the edges of the strains were light yellow.

#### 3.4.2. Antifungal Activity of Liquid Fermentation Extract

**Figure 4:**
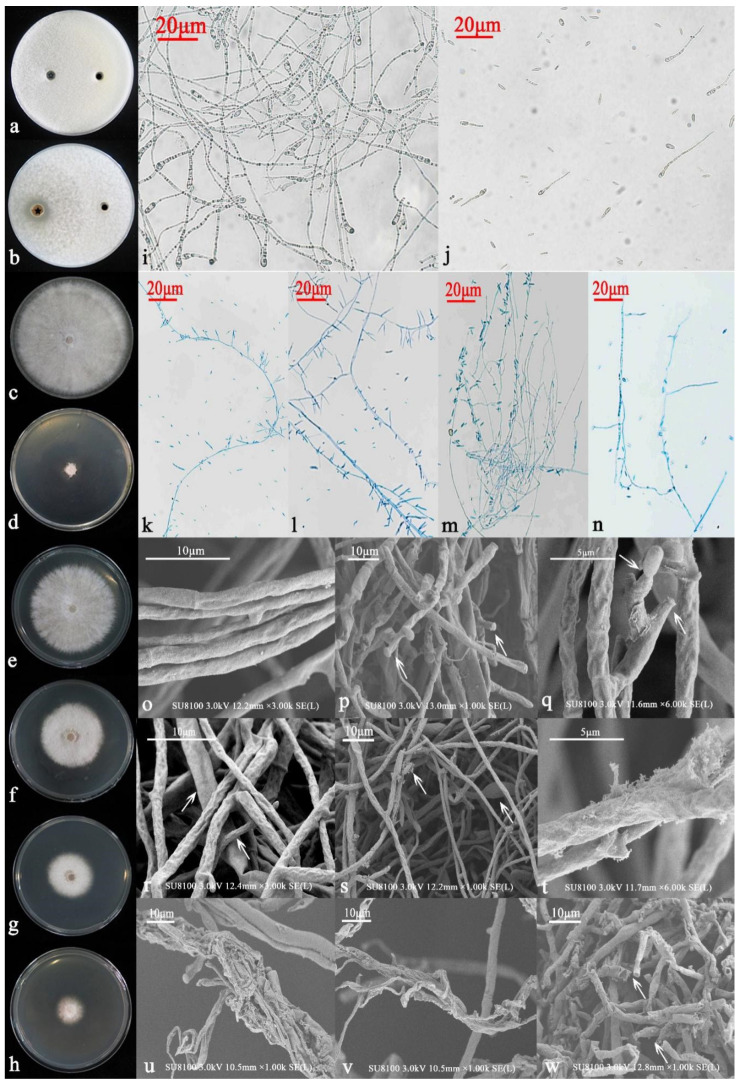
Antifungal activity of 6S-2 fermentation extract. Oxford cup test (**a**) with methanol on the left and sterile water on the right and (**b**) with 6S-2 fermentation extract on the left and sterile water on the right. (**c**) Cellophane assay performed with blank agar block for 7 d, followed by 7 d of MR5 culture. (**d**) Cellophane assay performed with 6S-2 for 7 d, followed by 7 d of MR5 culture. (**e**) MR5 cultivated for 7 d on PDA with 50 mg/L 6S-2 fermentation extract. (**f**) MR5 cultivated for 7 d on PDA with 100 mg/L 6S-2 fermentation extract. (**g**) MR5 cultivated for 7 d on PDA with 150 mg/L 6S-2 fermentation extract. (**h**) MR5 cultivated for 7 d on PDA with 200 mg/L 6S-2 fermentation extract. (**i**) Spore germination of MR5 after methanol addition to culture for 36 h (20× magnification). (**j**) Spore germination of MR5 after addition of 6S-2 fermentation extract to culture for 36 h (20× magnification). (**k**,**l**) MR5 fungal structures after 7 d of growth in the cellophane assay culture system using a blank agar block control (20× magnification). (**m**,**n**) MR5 fungal structures after 7 d of growth in the cellophane assay culture system previously inoculated with 6S-2 (20× magnification). (**o**) SEM observations of MR5 fungal structures after 7 d of growth in the cellophane assay culture system using a blank agar block control. (**p**–**w**) SEM observations of MR5 fungal structures after 7 d of growth in the cellophane assay culture system previously inoculated with 6S-2. The arrow indicates the specific and typical phenotypes described in the manuscript.

The 6S-2 fermentation broth that was extracted with ethyl acetate and re-dissolved in methanol, inhibited the growth and spread of MR5 hyphae, forming a transparent inhibition circle, and the sparse hyphae grew in the range of nearly 1/2 arc (Figure 4b). Seven days after cellophane was inoculated with MR5, the control cultures had overgrown the plate, and the hyphae were vigorous and dense (Figure 4c). When the cellophane was first inoculated with 6S-2 for 7 d, then removed, and the plate was inoculated with MR5, the horizontal expansion of the hyphae was almost zero. Sparse, sporadic hyphae were too long and grew vertically, indicating that some metabolites from 6S-2 inhibited the growth of MR5 (Figure 4d).

Next, the 6S-2 fermentation broth that was extracted with ethyl acetate and re-dissolved in methanol was mixed with PDA and diluted to different concentrations (50, 100, 150, and 200 mg/L). Treatment of MR5 with the broth extract inhibited the growth of MR5 hyphae to varying degrees (Figure 4e–h). As the extract concentration increased, the inhibition of hyphal growth increased and was found to be 11.52%, 36.43%, 59.11%, and 73.98% for the four extract concentrations (Supplementary Figure S4c). To explore the reasons behind this phenomenon, we performed spore germination assays and observed spores and hyphae by optical microscopy and SEM. After 36 h of methanol-only incubation, there were obvious white hyphae on the concave glass slide. Observed at 20× magnification, the germination rate of the MR5 spores reached 98%; the tails of the spores were relatively thick and entangled (Figure 4i). After incubation with the 6S-2 fermentation extract for 48 h, no obvious turbidity was detected on the concave glass slide. Observed at 20× magnification, the spore germination rate was only 15%, and the spore tails were relatively slender and grew slowly (Figure 4j). We then picked the edge hyphae and stained them with lactophenol cotton blue. Observed at 20× magnification, the MR5 control showed smooth single hyphae with sickle-shaped spores, clearly separated, multiple and full, with multiple hyphae arranged neatly. Hyphae were straight with no entanglement, and the spores were evenly arranged (Figure 4k,l). When 6S-2 was inoculated on the cellophane for 7 days, then removed, and MR5 was inoculated, the thickness of the individual MR5 hyphae was uneven, and some of the hyphae were curved or even broken (Figure 4m,n). Multiple hyphae were messy and entangled, with an uneven bending thickness. The spores were small, sparse, shriveled, and unevenly distributed. SEM observations of the MR5 controls showed smooth hyphae with obvious separation, uniform thickness, and no breaks. We observed no shrinkage, curling, deformation, damage, or spilling of contents (Figure 4o). By contrast, in the 6S-2 treatment, the MR5 hyphae shrank, the spores shriveled, the tips became blunt, and sickle-type spores became deformed. The growth points at the tips of the hyphae disappeared (Figure 4p). The spore tips were swollen, bifurcation was obvious, and some hyphae appeared damaged (Figure 4q). The hyphae were swollen, shrunken, bent, folded, and arranged unevenly; some portions were damaged. The hyphal tips were swollen, or parts of the hyphae were unevenly swollen and shrunken; the surface of the hyphae gradually became reticulated. Multiple hyphae were deformed, twisted, and entangled; holes appeared on the surface of the hyphae; some hyphae were broken, severely damaged, and began leaking their contents (Figure 4r–w).

#### 3.4.3. Antifungal Analysis of Volatile Substances

**Figure 5:**
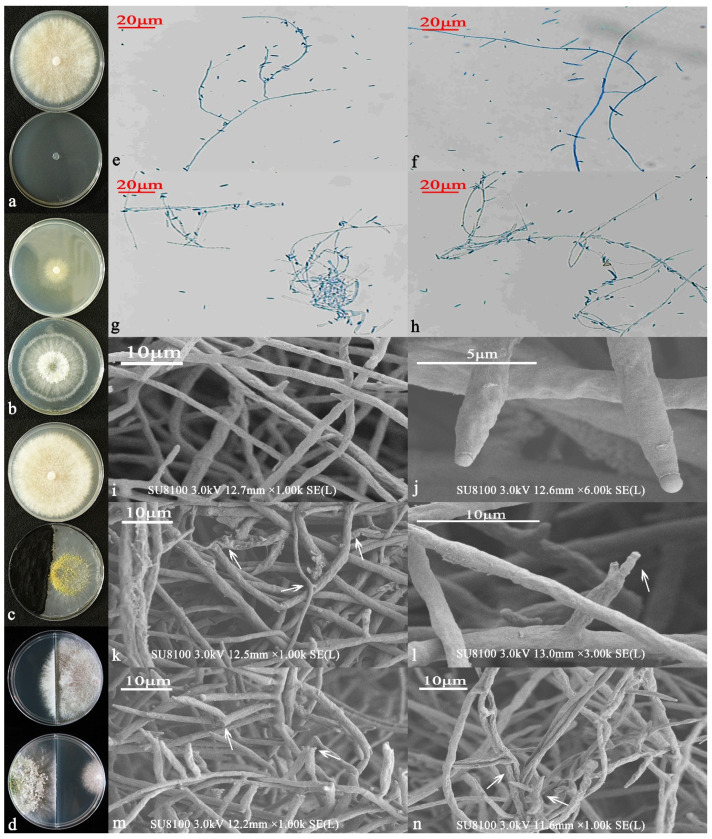
Antifungal activity of 6S-2 volatile substances. Sandwich plate test: (**a**) upper MR5, lower blank PDA medium, (**b**) upper MR5, lower 6S-2 PDA medium, (**c**) upper MR5, lower 6S-2 PDA medium and activated carbon. (**d**) MR5 control cultured in a two-part plate (top) and 6S-2 and MR5 cultured in a two-part plate (bottom). (**e**,**f**) Morphology of MR5 control hyphae observed at 20× magnification. (**g**,**h**) Morphology of MR5 hyphae after co-cultivation with 6S-2 observed at 20× magnification. (**i**,**j**) SEM observations of MR5 control hyphae. (**k**–**n**) SEM observations of MR5 hyphae after co-cultivation with 6S-2. The arrow indicated the specific and typical phenotypes described in the manuscript.

The test results revealed that volatile substances produced by 6S-2 significantly inhibited MR5 mycelial growth in the sandwich plate assay (Figure 5a,b), with an inhibition rate of 42.08%. The same results were obtained with the two-compartment Petri plate (90 mm), and the inhibition rate was 53.50% (Figure 5d). When 6S-2 was cultured with activated carbon, the growth of MR5 hyphae was accelerated and did not differ significantly from the control (Figure 5b,c). The control hyphae and buckle-cultured hyphae were selected for microscopy observation (20×). The MR5 controls showed evenly arranged hyphae that were smooth, with no obvious entanglement; the spores were sickle-shaped, evenly separated, large, and full (Figure 5e,f). After the co-cultivation of MR5 and 6S-2 on the two-part plate, the thickness of the MR5 mycelium was found to be uneven, with obvious bending, twisting, and breaking; the spores were small and shriveled (Figure 5g,h). SEM observations showed that the MR5 control hyphae were smooth and full, arranged evenly, and undamaged; the spore tips were round and full (Figure 5i,j). By contrast, hyphae inhibited by the 6S-2 volatile substances exhibited uneven thickness; they were twisted and entangled, part of their epidermis was broken and falling off, and began to leak their contents. The spore tips were shriveled with obvious bifurcations, breakage, and shedding. The mycelial tips were damaged, parts of the hyphae were broken, the contents of the epidermis leaked, and the hyphae were arranged in a disorganized manner. The hyphae were shriveled, twisted, coiled, and deformed (Figure 5k–n).

### 3.5. Arabidopsis thaliana Growth Promotion Test

**Figure 6:**
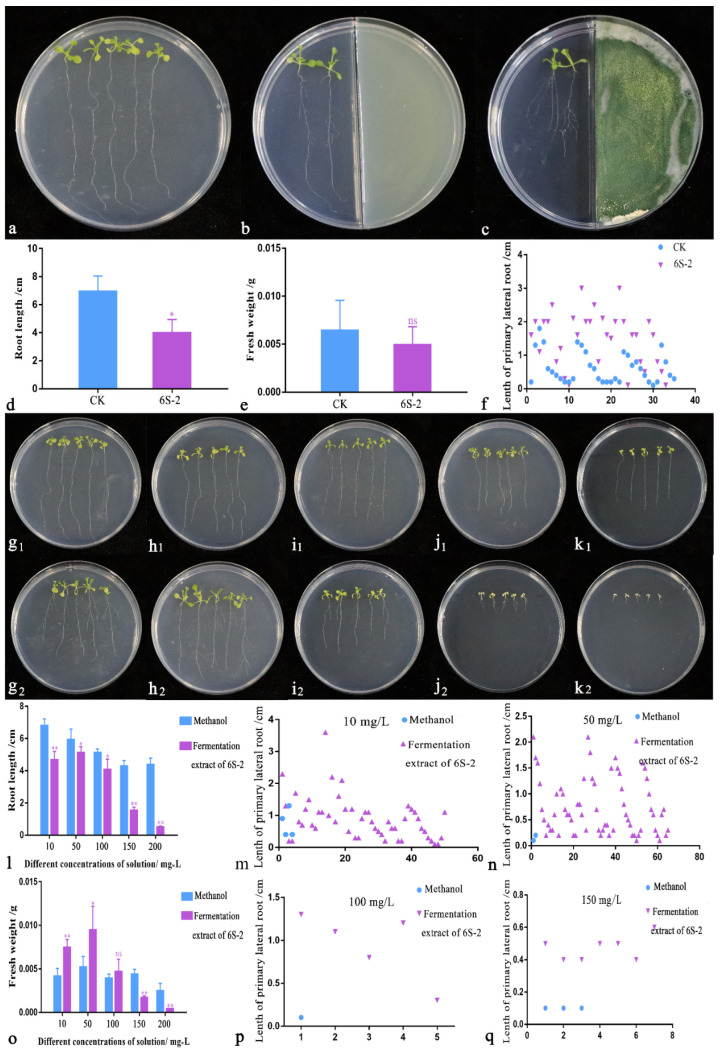
Effects of 6S-2 volatile substances and fermentation extract on *Arabidopsis thaliana* growth. (**a**) *Arabidopsis thaliana* seedlings grew vigorously on ½ MS medium. (**b**) *Arabidopsis thaliana* seedlings grew vigorously on a two-compartment Petri plate with ½ MS medium on the left and PDA blank medium on the right. (**c**) *Arabidopsis thaliana* grew vigorously when co-cultivated with 6S-2 on a two-part plate with ½ MS medium on the left and PDA medium inoculated with 6S-2 on the right. (**d**) *Arabidopsis thaliana* root lengths on the two-part plate. (**e**) Fresh weight of *Arabidopsis thaliana* on the two-part plate. (**f**) Number of primary lateral roots and the length distribution of lateral roots on the two-compartment Petri plate. (**g_1_**–**k_1_**) Growth of *Arabidopsis thaliana* on ½ MS medium with different concentrations of methanol solution: 10 mg/L (**g_1_**), 50 mg/L (**h_1_**), 100 mg/L (**i_1_**), 150 mg/L (**j_1_**), and 200 mg/L (**k_1_**). (**g_2_**–**k_2_**) Growth of *Arabidopsis thaliana* on ½ MS medium with different concentrations of 6S-2 fermentation extract: 10 mg/L (**g_2_**), 50 mg/L (**h_2_**), 100 mg/L (**i_2_**), 150 mg/L (**j_2_**), and 200 mg/L (**k_2_**). *Arabidopsis thaliana* root lengths at different concentrations of 6S-2 extract (**l**), fresh weight at different concentrations of 6S-2 extract (**o**), first-order lateral root numbers, and lateral root lengths at different concentrations of 6S-2 extract: 10 mg/L (**m**), 50 mg/L (**n**), 100 mg/L (**p**), and 150 mg/L (**q**). The significance of differences between groups was determined by *t*-test. * In each row of the same indicator represents significant differences between the methanol and fermentation extract of 6S-2 at *p* < 0.05; ** indicates extremely significant differences at *p* < 0.01; and ns indicates no significant differences between the methanol and fermentation extract of 6S-2.

When *Arabidopsis thaliana* was grown in a ½ MS medium without any added substances for 9 d, the root system grew vertically downwards, and very few lateral roots were produced (Figure 6a). When cultured on a two-part plate, the root system grew vertically downwards to approximately the same length as the medium. There were lateral roots on around half of the main roots, but the whole root system was shorter (Figure 6b). When the plants were co-cultured with the 6S-2 spore solution, the root system was shorter and only grew to around 2/3 the length of the medium. However, lateral-root production was markedly stimulated, and lateral-root length was three times higher than that of the controls (Figure 6c,f). There were no significant differences in fresh weight between the control and 6S-2-treated seedlings (Figure 6e). Compared with the blank control, the addition of different concentrations of methanol to ½ MS medium (10–200 mg/L) suppressed the *Arabidopsis thaliana* root length in a dose-dependent manner, but the number and length of lateral roots did not differ significantly. Compared with methanol addition, the addition of the 6S-2 extract altered the *Arabidopsis thaliana* root length to varying degrees (Figure 6(g1–k1,g2–k2,g3–k3)). The *Arabidopsis thaliana* root length, number of lateral roots, and lateral root length were highest at 50 mg/L 6S-2 fermentation extract (Figure 6(h2,l,n)). As the extract concentration increased from 50 to 200 mg/L, the root length gradually decreased, but the number of lateral roots and the length of lateral roots increased to varying degrees. For 10–100 mg/L of 6S-2 fermentation extract, the fresh weight of *Arabidopsis thaliana* was higher than that of the corresponding methanol controls. At 200 mg/L, root-system growth was significantly inhibited, the whole plant was yellow-white, and the leaves became thicker and harder (Figure 6(k2)).

### 3.6. GC-MS Analysis

After liquid sampling, a GC-MS analysis, and a comparison of the spectra with the NIST 17 database and reference literature, 283 substances were identified, 44 of which had relative peak areas greater than 0.5. These included 10 alcohols, 8 alkanes, 8 ketones (including 5 furanones), 5 acids, 5 phenols, 1 ester, 1 olefin, 1 ether, and 5 other substances (Supplementary Table S5, Supplementary Figure S5). However, after direct collection by headspace extraction and the removal of components that overlapped with the PDA blank, 201 substances were identified, and only 18 had relative peak areas greater than 0.5. These included 6 alkanes, 4 alcohols, 2 ketones, 1 furan, and 5 other substances. The largest relative peak area was identified for 6-pentyl-2H-pyran-2-one (6-PP) (36.45%); this volatile substance has been reported to have antifungal and growth-promoting functions (Table 1, Supplementary Figure S6).

### 3.7. Effects of 6S-2 Fertilizer Containing Spore Powder in Pot Experiments

**Figure 7:**
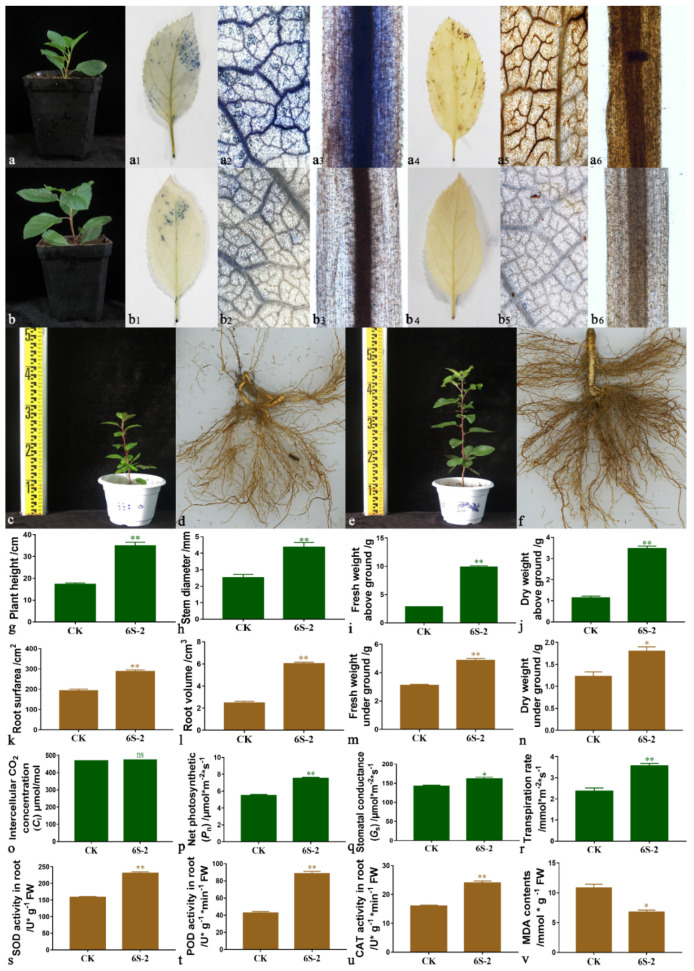
Growth measurements, root protective enzyme activities, and photosynthetic indexes in pots. (**a**) T337 in replant soil; (**b**) T337 in replant soil with 6S-2 spore fertilizer; (**c**) *M. hupehensis* in replant soil; (**d**) Root scan of *M. hupehensis* in replant soil; (**e**) *M. hupehensis* in replant soil with 6S-2 spore fertilizer; (**f**) Root scan of *M. hupehensis* in replant soil with 6S-2 spore fertilizer; NBT staining of leaves (**a_1_**,**a_2_**,**b_1_**,**b_2_**) and roots (**a_3_**,**b_3_**); DAB staining of leaves (**a_4_**,**a_5_**,**b_4_**,**b_5_**) and roots (**a_6_**,**b_6_**); Plant height of *M. hupehensis* (**g**); Stem diameter of *M. hupehensis* (**h**); Shoot fresh weight of *M. hupehensis* (**i**); Shoot dry weight of *M. hupehensis* (**j**); Root surface area (**k**); Root volume (**l**); Root fresh weight of *M. hupehensis* (**m**); Root dry weight of *M. hupehensis* (**n**); Ci (**o**); *P*_n_ (**p**); *G*_s_ (**q**); *T*_r_ (**r**); SOD (**s**); POD (**t**); CAT (**u**); MDA (**v**). The significance of differences between groups was determined by *t*-test. * In each row of the same indicator represents significant differences between the replant soil with 6S-2 spore fertilizer and control at *p* < 0.05; ** indicates extremely significant differences at *p* < 0.01; and ns indicates no significant differences between the replant soil with 6S-2 spore fertilizer and control.

The growth of T337 seedlings was improved by the application of 6S-2 spore fertilizer (Figure 7b); the coverage area of the NBT stain on leaf edges was reduced (Figure 7(b1)), and the degree of staining was reduced (Figure 7(b2)). The same results were obtained in roots after NBT staining (Figure 7(b3)). These results showed that O2^−^ accumulation in tissues was reduced, and that plants’ antioxidant capacity was improved. DAB-staining results showed that a large amount of peroxide was concentrated at the leaf edges and in the roots of control seedlings (Figure 7(a4–a6)), and oxidative damage appeared to be severe. Further pot-experiment results showed that 6S-2 spore fertilizer application promoted the growth of *M. hupehensis* seedlings (Figure 7c–n), which differed significantly from that of controls. The application of 6S-2 also increased the net photosynthetic efficiency (Figure 7p), root protective enzyme activities (Figure 7s–u), and reduced MDA content (Figure 7v). The amount of fungi in the soil (especially the *Fusarium* copy number) was also significantly reduced, but the number of culturable bacteria increased, causing a significant increase in the ratio of bacteria to fungi (Supplementary Figure S7).

## 4. Discussion

*Trichoderma* displays a good antagonistic ability against a variety of plant pathogens, inhibiting their hyphal growth and spore germination. This broad-spectrum of antifungal activity greatly relies on Trichoderma’s competition for ecological space and its strong parasitic and antibiotic mechanisms, including both volatile and non-volatile secondary metabolites [23,24,25,26,27,65]. In this experiment, 6S-2 was identified as an endophyte in the root systems of apples; its molecular and morphological characteristics confirmed that it was *T. asperellum.* Furthermore, 6S-2 exhibited the same broad-spectrum antifungal effect previously demonstrated in other *Trichoderma* spp. It grew rapidly, quickly produced large numbers of conidia, and was able to cover the entire medium within 5 d, occupying the growth space of pathogenic fungi and slowing their hyphal growth. It also showed strong amylase activity, which has been demonstrated to enhance the competitiveness of *T. asperellum* [66,67], but whether the nutrients required for pathogen growth are consumed during its rapid growth process requires further study. Additionally, 6S-2 showed activity of hydrolytic enzymes such as cellulase and protease; proteases were able to break down proteins in the cell walls of pathogenic fungi into peptide chains and constituent amino acids, accelerating the parasitic ability of 6S-2 on pathogenic fungi. This allows the mycelium to directly enter the cavity of the target fungus, causing the pathogen to grow slowly or even dissolve and die [68], thus destroying its pathogenicity toward the plant. The secretion of laccase can also enhance the hyphal growth of *T. asperellum*. When *Trichoderma* antagonizes pathogenic fungi alone, the defense tolerance of *Trichoderma* promotes its parasitic process [67], and the production of chlamydospores can enhance the ability of *Trichoderma* to resist harsh environments and maintain normal growth [69].

When MR5 was inoculated on cellophane after 7 d of 6S-2 growth, the growth of MR5 was significantly inhibited, and its hyphae were twisted, swollen, and ruptured, indicating that the secondary metabolites of 6S-2 had antifungal properties. The liquid fermentation product was extracted and formulated at different concentrations, and a similar antifungal effect was achieved. In addition, the sandwich-panel test showed that volatile substances produced by 6S-2 could also inhibit pathogen growth, which was consistent with previous research results [70], confirming that *Trichoderma* volatiles have negative effects on pathogenic fungi [71]. The analysis indicated that secondary metabolite alkanes, alcohols, furans, and furanones produced by *T. asperellum* 6S-2 have strong antifungal effects [71,72] and may act as precursors for other antifungal compounds that play a more important role through a synergistic effect. They may also induce a defensive signal cascade [62]. In addition, studies have shown that 6-PP, which is a specific volatile produced by *Trichoderma*, has strong antifungal activity [73]. It inhibits the growth of pathogenic fungi by inhibiting spore germination [71].

Our results show that volatiles produced by 6S-2 could promote the development of *Arabidopsis thaliana* lateral roots, and that the addition of 50 mg/mL of the fermentation extract produced the greatest lateral root numbers and lengths. Studies have shown that *Trichoderma* promotes plant growth by dissolving nutrients through rhizosphere acidification, producing auxin and secondary metabolites, and emitting mixed volatiles [74,75]. An analysis suggested that co-cultivation with 6S-2 may directly and indirectly promote the growth and development of *Arabidopsis thaliana*. First, 6S-2 could directly produce plant-growth-promoting chemicals, such as IAA, siderophores, and ammonia, and it could dissolve phosphorus and potassium. The plant growth hormone, IAA, can promote lateral root growth and increase biomass; within a certain concentration range, the effects of IAA on the biomass and lateral root growth and development of *Arabidopsis thaliana* correlate positively [69]. Secreted siderophores can combine with insoluble iron (Fe^3+^) and convert this into easily absorbable Fe^2+^, which participates in the synthesis of various enzymes, thereby promoting plant growth and development [76]. The presence of ammonia can promote plant-nitrogen accumulation and the production of 1-aminocyclopropane-1 carboxylic acid deaminase (ACC deaminase), which regulates ethylene production. The presence of ammonia can promote the growth and development of *Arabidopsis thaliana* [77,78]. In addition, phosphate solubilization and potassium released by 6S-2 may stimulate the plant immune system and provide essential nutrients for the growth and development of *Arabidopsis thaliana* [79]. Second, volatiles can also promote plant growth and development, and act as signaling molecules to activate auxin and/or other plant growth hormone-related pathways [80]. Volatiles can enhance plant vitality and promote lateral-root development through the production of antibiotics, the suppression of plant pathogens, and the induction of defense responses [81,82].

Volatile substances can diffuse readily and can act as inducers of defense and development during the process of plant–fungal interaction, activating signal transduction cascades to promote appropriate responses [71]. However, in this experiment, the root length of *Arabidopsis thaliana* was suppressed to some extent when co-cultured with 6S-2, compared with the control. These different results may reflect the use of different *Trichoderma* species with different volatile profiles in different media. The volatile concentrations produced in the culture device may also have differed from those in previous experiments. If the distance between the *Arabidopsis thaliana* seedlings and the fungus was too great, the concentrations of root-elongation-promoting compounds may have been too low; they may not have reached the root tip to stimulate auxin precursors. Alternatively, the synthesis of substances involved in the auxin signaling pathway may have been inhibited [79]. Instead, *Arabidopsis thaliana* may have regarded *Trichoderma* volatiles and high-concentration extracts as stress compounds and may have, as a result, initiated multiple levels of response (morphological, physiological, and transcriptional) and signaling cascades to resist this perturbation [55,83], thereby inhibiting the elongation of the main root and the development of lateral roots. This result was consistent with the inhibition of the primary root growth of *Arabidopsis thaliana*, leaf size, and fresh weight by *Trichoderma* volatiles, reported in Cottier and Mühlschlegel [84]. In addition, volatile organic compounds produced by *Trichoderma* in the process of division and interaction can induce the accumulation and redistribution of auxin in roots, and 6-PP not only displays antifungal activity but also acts as a growth regulator in lateral root primordia, especially by inducing auxin [82,83]. Through its regulation of ethylene signal transduction and auxin transport [85], 6-PP directly affects plant biomass production and root architecture but it can inhibit taproot growth at high concentrations [79,82].

Although we have verified the antifungal and growth-promoting effects of *Trichoderma* to some extent in this work, the molecular mechanisms by which 6S-2 inhibits MR5, a *Fusarium* strain associated with ARD, remain unknown. In addition, *Trichoderma* is a traditional biocontrol bacterium. To develop its use for comprehensive disease management, further tests should also evaluate its compatibility with chemical fungicides. Its bioremediation ability for environmental pollution should also be explored in order to promote the eco-friendly management of diseases. At present, most *Trichoderma* products used for biological control are live fungal preparations, and there have been fewer studies on *Trichoderma* metabolites. Live fungal preparations have a short shelf life and cannot effectively and stably exert antifungal effects. This antifungal substance could be enriched by organic solvent extraction and concentration for biomimetic synthesis and used in the development of new biological pesticides. There are probably many other undiscovered fungal parasitic-related enzymes, elicitors, and primary and secondary metabolites in Trichoderma, and these will be the focus of future experiments.

## 5. Conclusions

The endophytic antagonistic fungus 6S-2 was isolated from the roots of nine healthy apple trees in replanted apple orchards and identified as *T. asperellum*, based on morphological traits and sequence analysis. We found that 6S-2 has protease, amylase, cellulase, and laccase activities, and that it can secrete active substances such as iron carriers, auxin, and ammonia. It is capable of dissolving phosphorus and potassium. Furthermore, 6S-2 exhibits strong fungistatic activity against the apple-specialized *Fusarium proliferatum* MR5. It also shows broad-spectrum antagonistic activity towards multiple pathogenic fungi. Both the volatile substances and the fermentation extract of 6S-2 can inhibit the growth of MR5 mycelium to a certain extent, and can increase the number and elongation of *Arabidopsis thaliana* lateral roots. In pots, the application of 6S-2 fertilizer can reduce oxidative damage to T337 leaves and roots, promote photosynthesis, and enhance the activities of root protective enzymes, thereby promoting plant growth.

## Figures and Tables

**Table 1:** Composition analysis of volatile secondary metabolites from 6S-2.

Retention Time	Area (%)	Possible Compounds	Molecular Formula	CAS Number
23.341	36.45	2H-Pyran-2-one, 6-pentyl-	C_10_H_14_O_2_	27593-23-3
11.081	7.09	Cyclotrisiloxane, hexamethyl-	C_6_H_18_O_3_Si_3_	541-05-9
14.831	6.43	Furan, 2-pentyl-	C_9_H_14_O	003777-69-3
14.549	3.34	Cyclotetrasiloxane, octamethyl-	C_8_H_24_O_4_Si_4_	556-67-2
8.73	2.68	Silanediol, dimethyl-	C_2_H_8_O_2_Si	1066-42-8
8.766	2.6	Silanediol, dimethyl-	C_2_H_8_O_2_Si	1066-42-8
23.978	2.51	Butylated Hydroxytoluene	C_15_H_24_O	128-37-0
20.65	2.42	Cyclohexasiloxane, dodecamethyl-	C_12_H_36_O_6_Si_6_	540-97-6
15.535	2.38	1-Hexanol, 2-ethyl-, (S)-	C_8_H_18_O	128821-84-1
17.613	2.31	Cyclopentasiloxane, decamethyl-	C_10_H_30_O_5_Si_5_	541-02-6
9.659	1.65	1-Pentanol	C_5_H_12_O	71-41-0
23.267	1.16	Cycloheptasiloxane, tetradecamethyl-	C_14_H_42_O_7_Si_7_	107-50-6
16.778	1.15	2,5-Dihydroxybenzaldehyde	C_7_H_6_O_3_	1194-98-5
12.611	1.06	Oxime-, methoxy-phenyl-_	C_8_H_9_NO_2_	-
25.474	0.95	Cyclooctasiloxane, hexadecamethyl-	C_16_H_48_O_8_Si_8_	556-68-3
13.704	0.94	4′,6′-Dimethoxy-2′,3′-dimethylacetophenone	C_12_H_16_O_3_	-
24.469	0.52	Octanal, 7-methoxy-3,7-dimethyl-, (S)-	C_11_H_22_O_2_	134678-53-8
24.748	0.51	Cyclobarbital	C_12_H_16_N_2_O_3_	52-31-3

## Supplementary Materials

The following are available online at https://www.mdpi.com/article/10.3390/jof7121050/s1, Figure S1: Solid fermentation process of *T. asperellum* 6S-2 in shallow plate. a: Fermentation substrate loading, and fermentation start; b: *T. asperellum* (6S-2) hyphae formation; c: *T. asperellum* (6S-2) spore formation; d: Fermentation finish; e: Spore powder after crushing and sieving. Figure S2: Principal component analysis (PCA) of the T-RFLP profiles from healthy (H) and unhealthy (U) soils from MWW (a), MWL (b), MGD (c), QGP (d), QYC (e), YZY (f), YXX (g), LSW (h), and LSF (i). T-RFLP profiles were obtained following digestion of soil DNA using the Hha I enzyme. MWW: Wanggezhuang Village, Wanggezhuang Town, Muping District, Yantai City, Shandong Province; MWL: Luanjiatuan Village, Wanggezhuang Town, Muping District, Yantai City, Shandong Province; MGD: Dapankou Village, Guanshui Town, Muping District, Yantai City, Shandong Province; QGP: Panjialing Village, Guanli Town, Qixia City, Shandong Province; QYC: Changjiagou Village, Yangchu Town, Qixia City, Shandong Province; YZY: Yangjiazhuang Village, Zhongzhuang Town, Yiyuan County, Zibo City, Shandong Province; YXX: Xinzhuang Village, Xili Town, Yiyuan County, Zibo City, Shandong Province; LSW: Wantou Village, Shahe Town, Laizhou City, Shandong Province; LSF: Fengmaozhai, Shahe Town, Laizhou City, Shandong Province; Figure S3: Maximum-likelihood phylogenetic tree of the combined ITS and TEF genes from 44 taxa of Trichoderma. Sequences of Protocrea pallida and Protocrea farinosa were used as outgroups. Bootstrap values ≥70% and Bayesian posterior probabilities ≥0.90 are shown. The scale bar represents 10 substitutions per nucleotide position. The sequence from the fungal species obtained in this study is presented in bold and is identical to that of the known species *T. asperellum* SZMC:24288; Figure S4: Effects of 6S-2 volatile substances and different concentrations of liquid fermentation extract on the growth radius of MR5 hyphae. (a) Hyphal radius with control or 6S-2 volatile substances in the two-part plate assay. ** *p* < 0.001 (n = 3, Student’s *t*-test). (b) Hyphal radius with control or 6S-2 volatile substances in the sandwich plate assay. Ac, activated carbon. (c) Hyphal radius with different concentrations of 6S-2 fermentation extract. Bars with different letters are significantly different, *p* < 0.05 (n = 3; one-way ANOVA and Tukey’s mean separation test); Figure S5: Mass spectrum of SPME-GC-MS; Figure S6: Mass spectrum of HS-SPME-GC-MS; Figure S7: The soil culturable microorganisms: bacteria (a); fungi (b); actinomycetes (c); and bacteria/fungi ratio (d); the real-time fluorescence quantification of four *Fusarium* species: *F. oxysporum* (e); *F.* *proliferatum* (f); *F. solani* (g); and *F. moniliforme* (h); Table S1: The main soil physicochemical properties of nine apple orchards in the Yantai and Yiyuan regions of China. HN: hydrolyzed nitrogen; AP: available phosphorus; AK: available potassium; SOM: soil organic matter.; Table S2: The detailed for specific formula of the media used in this manuscript; Table S3: The specific procedures of GC-MS analysis; Table S4: ITS and TEF gene accession numbers for 44 strains used in the construction of the phylogenetic tree; Table S5: Composition analysis of secondary metabolites in 6S-2 fermentation liquid.
