# Supplementary material for: The Endophytic Strain Trichoderma asperellum 6S-2: An Efficient Biocontrol Agent against Apple Replant Disease in China and a Potential Plant-Growth-Promoting Fungus
Source: J Fungi (Basel). 2021 Dec 8;7(12):1050. doi: 10.3390/jof7121050 (PMC8705406; doi:10.3390/jof7121050)
Supplement: Supplementary file 1 [file jof-07-01050-s001.zip › jof-1474129-supplementary.pdf]

Supplementary Tables

For "The endophytic strain Trichoderma asperellum 6S-2: An efficient biocontrol agent against apple replant disease in China and a potential plant-growth-promoting fungus"

Supplementary legends

Supplementary Table S1 The main soil physicochemical properties of nine apple orchards in the Yantai and Yiyuan regions of China

Supplementary Table S2 The detailed for specific formula of the media used in this manuscript

Supplementary Table S3 The specific procedures of GC-MS analysis

Supplementary Table S4 ITS and TEF gene accession numbers for 44 strains used in the construction of the phylogenetic tree

Supplementary Table S5 Composition analysis of secondary metabolites in 6S-2 fermentation liquid

Supplementary Table S1

Areas	HN (mg/kg)	AP (mg/kg)	AK (mg/kg)	SOM (g/kg)	PH	Planting years	Replanted years	Rootstocks	Scion	Coordinate	Soil texture
MWW	68.64	114.49	270.47	15.70	6.67	15	4	*Malus. Robusta.*	Fuji	37.17N,121.41E	Clay loam
MWL	43.70	60.97	321.19	6.39	6.21	25	5	*Malus. Robusta.*	Fuji	37.44N,121.18E	Sandy loam
MGD	54.76	75.54	352.30	9.73	5.46	20	3	*Malus. Robusta.*	Fuji	37.21N,121.25E	loam
QGP	59.84	142.71	262.94	16.78	4.90	18	4	*Malus. Robusta.*	Yanfu No.3	37.20N,126.76E	Silt clay loam
QYC	45.24	61.65	318.80	6.67	5.23	18	4	*Malus. Robusta.*	Gala	37.21N,120.80E	Sandy loam
YZY	23.98	48.15	155.15	9.46	5.24	20	5	*Malus. Robusta.*	Fuji	36.08N,118.26E	Clay loam
YXX	34.89	57.01	203.82	8.68	5.08	20	5	*Malus. Robusta.*	Fuji	36.00N,118.22E	Clay loam
LSW	29.53	71.55	297.08	6.60	6.46	20	4	*Malus. Robusta.*	Yanfu No.3	37.08N,119.79E	Clay loam
LSF	33.97	96.50	123.49	5.84	4.57	20	4	*Malus. Robusta.*	Yanfu No.3	37.41N,120.06E	loam

Note: HN: hydrolyzed nitrogen; AP: available phosphorus; AK: available potassium; SOM: soil organic matter. MWW: Wanggezhuang Village, Wanggezhuang Town, Muping District, Yantai City, Shandong Province; MWL: Luanjiatuan Village, Wanggezhuang Town, Muping District, Yantai City, Shandong Province; MGD: Dapankou Village, Guanshui Town, Muping District, Yantai City, Shandong Province; QGP: Panjialing Village, Guanli Town, Qixia City, Shandong Province; QYC: Changjiagou Village, Yangchu Town, Qixia City, Shandong Province; YZY: Yangjiazhuang Village, Zhongzhuang Town, Yiyuan County, Zibo City, Shandong Province; YXX: Xinzhuang Village, Xili Town, Yiyuan County Zibo City, Shandong Province; LSW: Wantou Village, Shahe Town, Laizhou City, Shandong Province; LSF: Fengmaozhai, Shahe Town, Laizhou City, Shandong Province.

Supplementary Table S2

Media names	Formula	References
Potato dextrose agar (PDA) medium	200.0 g peeled potatoes, 20.0 g glucose, and 20.0 g agar in 1 L distilled water, then heating at 121℃ for 20 min	[86]
PDB medium	200.0 g peeled potatoes, and 20.0 g glucose, in 1 L distilled water, then heating at 121℃ for 20 min	[86]
Charpy medium	2.0 g KNO_3, 1.0 g K_2HPO_3, 1.0 g KCl, 0.5 g $MgSO_4·7H_2O$, 0.01 g $FeSO_4$, 30.0 g sucrose, and 20.0 g agar in distilled water to a volume of 1 L, then heating at 121°C for 20 min	[87]
CMD medium	50.0 g corn flour, 2.0 g glucose, and 15.0 g agar in distilled water to 1 L (final pH 6.0), then heating at 121℃ for 20 min	[88]
Saltwater nutrient agar (SNA) medium	1.0 g KH_2PO_4, 0.5 g KCl, 1.0 g KNO_3, 0.5 g $MgSO_4$, 0.2 g glucose, 0.2 g sucrose, and 18.0 g agar in distilled water to 1 L, then heating at 121℃ for 20 min	[88]
Phosphate broth (NBRIP)medium	10 g glucose, 10 g tricalcium phosphate, 5.0 g $MgCl_2·6H_2O$, 0.25 g $MgSO_4·7H_2O$, 0.2 g KCl, 0.1 g $(NH_4)_2SO_4$, and 20.0 g agar in distilled water to 1 L, then heating at 121℃ for 20 min	[89]
Amylase medium	20.0 g starch, 0.5 g KCl, 2.0 g $NaNO_3$, 1.0 g K_3PO_4, 0.5 g $MgSO_4·7H_2O$, 5.0 g NaCl, and 18.0 g agar in distilled water to 1 L, then heating at 121℃ for 20 min. When the colony had been cultivated, it was placed on the medium and covered with iodine solution	[68]
Chromeazurol S (CAS) medium	60.5 mg CAS, 72.9 mg hexadecyltrimethylammonium bromide (CTAB), 30.24 g piperazine-1,4-bis (2-ethanesulfonic acid) (PIPES), and 1 mM $FeCl_3·6H_2O$ in 10 mM HCl and agarose (0.9% w/v). 6S-2 was grown on PDA plates at 28°C for 7 d. Ten millilitre overlays of chromeazurol S (CAS) medium were applied to the plates, and a change in color of the overlaid medium after 2 h of incubation was indicative of siderophore production	[90-91]
Laccase medium	200.0 g peeled potatoes, 20.0 g glucose, 20.0 g agar, and 0.4 g guaiacol in 1 L distilled water, then heating at 121℃ for 20 min	[67]
Cellulase culture medium	2.0 g $(NH_4)_2SO_4$, 0.5 g $MgSO_4·7H_2O$, 1.0 g	[68]

	K_2HPO_4, 0.5 g NaCl, 2.0 g sodium carboxymethylcellulose, 0.4 g Congo red, and 22.0 g agar in distilled water to 1 L, then heating at 121°C for 20 min	
Protease culture medium	1% agar and 5% skimmed milk powder by volume, heating at 115°C for 30 min, then mixing and pouring the plate	[54]
Ammonia production medium	6S-2 was inoculated into a test tube that contained 10 mL of 4% peptone broth in a liquid medium, then cultured at 28±0.1°C. After 6–7 d of culture, 1 mL of Nesler reagent was added, and a color change from brown to dark brown was indicative of ammonia production	[92]
Indole acetic acid (IAA) production medium	6S-2 was grown in PDB amended with tryptophan (1 g L^{-1}) at 25°C for 7 d. Then 1 mL of filtrate was mixed with 2 mL of Salkowski reagent (2% 0.5 M $FeCl_3$ in 35% perchloric acid) at room temperature for 20 min, and the color change was observed	[92]
Half-strength Murashige and Skoog (MS) medium	Qingdao Hope Bio-Technology Co., Ltd. (Product number HB8469-6).	

Supplementary Table S3

	SPME-GC-MS	HS-SPME-GC-MS
Rtx-5MS column	60.0 m × 0.25 mm × 0.25 µm	60.0 m × 0.25 mm × 0.25 µm
Carrier gas	He (99.999%)	He (99.999%)
Carrier gas pressure	Initial: 500–900 kPa; maintained: 117.6 kPa	117.6 kPa
Column flow rate	1.0 mL/min	1.0 mL/min
Purge flow rate	3.0 mL/min	1.0 mL/min
Split ratio	10.0	1.0
Column oven temperature	25°C	50°C
Injection port temperature	250°C	250°C
Injection volume	1 µL	-
Injection time	-	1 min
Temperature program	50 °C for 2 min, raised to 180°C at 10°C/min, held for 1 min, raised to 300°C at 6°C/min, and held for 5 min.	50°C for 2 min, increased to 180°C at 8°C/min, increased to 250°C at 10°C/min, and held at 10 min.
Total program time	41 min	31.25 min
Sampling method	Automatic	Manual extraction handle (Supelco, USA)
Mass spectrometry conditions	Q3 Scan acquisition mode, 1250	Q3 Scan acquisition mode, relative

	u/sec scanning speed, 0.5 sec interval, 0.1 kV detector voltage, and the use of relative values	value EMV mode
Full scan acquisition mass range	45–650 m/z	50–550 m/z
Ion source EI	70 eV, and the interface temperature was 280 ℃, the ion source temperature was 200 ℃, and the solvent delay time was 3 min.	70 eV, and a temperature of 230°C

Supplementary Table S4

**Strain name**	**Strain number**	**GenBank Login ID**	
		**ITS 1,2**	**TEF1**
Trichoderma chlamydosporicum	HMAS 248850	KY687933.1	KY688052.1
Trichoderma estonicum	CBS 111147	FJ860752.1	FJ860638.1
Trichoderma harzianum	M110	MN555334.1	MN557854.1
Trichoderma strictipilis	HMAS 252545	KF923301.1	KF923285.1
Trichoderma hainanense	HMAS:248837	KY687920.1	KY688033.1
Trichoderma harzianum	HZA11	MH624146.1	MK850833.1
Trichoderma atroviridis	CBS 119499	FJ860726.1	FJ860611.1
Trichoderma afroharzianum	GJS 04-193	FJ442233.1	FJ463298.1
Trichoderma breve voucher	HMAS:248844	KY687927.1	KY688045.1
Trichoderma harzianum	CBS 226.95	AY605713	AY605833.1
Trichoderma pleuroti	CBS 124387	HM142363.1	HM142382.1
Trichoderma citrinoviride	GJS 92-8	KR812111.1	JN175595.1
Trichoderma citrinoviride	DUCC001	JF700484.1	JF700485.1
Trichoderma ghanense	18ASMA011	MT520631.1	MT671930.1
Trichoderma longibrachiatum	Tl35	MW191751.1	MW201701.1
Trichoderma longibrachiatum	Tl41	MW191752.1	MW201702.1
Trichoderma longibrachiatum	TR5	KC859426.1	KC572116.1
Trichoderma longibrachiatum	124L	KF889068.1	KF889260.1
Trichoderma rossicum	GJS 07-72	HQ342416.1	HQ342222.1
Trichoderma rossicum	GJS 98-89	HQ342420.1	HQ342225.1
Trichoderma semiorbis	GJS 99-108	HM466664.1	JN133576.1
Trichoderma brevicompactum	CBS 112447	EU330942.1	EU338300.1
Trichoderma brevicompactum	CBS 112443	EU330943.1	EU338281.1
Trichoderma parapiluliferum	CBS 120921	FJ860799.1	FJ179578.1
Trichoderma polysporum	T50	KX632525.1	KX632639.1
Trichoderma piluliferum	CBS 120927	FJ860810.1	FJ860674.1
Trichoderma hamatum	GJS 05-262	EU856292.1	EU856317.1
Trichoderma hamatum	GJS 05-334	EU856291.1	EU856316.1
Trichoderma viridescens	CBS 433.34	MH855595.1	AF456905.1
Trichoderma viridescens	TRS573	KP009373.1	KP008928.1

Trichoderma virilente	DAOM 234234	EU280119.1	EU280009.1
Trichoderma paraviridescens	T124	MT187973.1	MT214331.1
Trichoderma paraviridescens	FP-027-C5	MH102082.1	MH102123.1
Trichoderma neokoningii	CBS 120070	MH863076.1	KJ665620.1
Trichoderma gamsii	TW20050	KU523894.1	KU523895.1
Trichoderma gamsii	GJS 05-111	DQ841730.1	DQ841722.1
Trichoderma paratroviride	SFC102188	MF186130.1	MF185938.1
Trichoderma atroviride	CBS 142.95	MH862505.1	AY376051.1
Trichoderma caerulescens	S206	JN715590.1	JN715624.1
Trichoderma caerulescens	S195	JN715589.1	JN715621.1
Protocrea pallida	CBS 121552	EU703922.1	EU703897.1
Protocrea farinosa	CBS 121551	EU703910.2	EU703889.1
Trichoderma asperellum	SZMC:24288	MN516477.1	MN520032.1

Supplementary Table S5

Retention time	Area (%)	Possible Compounds	Molecular formula	CAS Number
6.775	6.99	Dibutyl phthalate	$C_{16}H_{22}O_4$	84-74-2
7.598	5.31	2(3H)-Benzofuranone, 3a,4,5,7a-tetrahydro-3a,6-dimethyl-, (3aR-cis)-	$C_{10}H_{14}O_2$	119479-03-7
8.118	4.76	Cubenol	$C_{15}H_{26}O$	21284-22-0
8.541	3.51	(R, Z)-2-Methyl-6-(4-methylcyclohexa-1,4-dien-1-yl) hept-2-en-1-ol	-	-
11.039	3.03	Hordenine	$C_{10}H_{15}NO$	62493-39-4
15.001	2.83	2(3H)-Benzofuranone, 3a,4,5,7a-tetrahydro-3,6-dimethyl-	$C_{10}H_{14}O_2$	57743-63-2
17.563	2.62	Farnesene epoxide, E-	$C_{15}H_{24}O$	-
18.061	2.25	Phytol	$C_{20}H_{40}O$	150-86-7
18.302	2.14	Sesquicineole	$C_{15}H_{26}O$	-
18.529	2.09	1-octeno-3-ol	$C_{10}H_{18}O_2$	2442-10-6
18.721	1.89	Norreticuline, N-formyl-	-	-
19.084	1.67	1H-Isoindole-1,3(2H)-dione, 2-[(4,5-dimethyl-2-furanyl) methyl]-	$C_{15}H_{13}NO_3$	63272-05-9
19.442	1.53	2H-Pyran-2-one, tetrahydro-4-hydroxy-6-pentyl-	$C_{10}H_{18}O_2$	705-86-2
19.646	1.49	2,4-Di-tert-butylphenol	$C_{14}H_{22}O$	96-76-4
19.796	1.38	Phloroglucitol	$C_6H_{12}O_3$	2041-15-8
19.858	1.29	Hexadecane	$C_{16}H_{34}$	544-76-3
20.063	1.26	beta. - Santalol	$C_{15}H_{24}O$	37172-32-0
20.117	1.23	6-(p-Tolyl)-2-methyl-2-heptenol, trans-	-	-
20.437	1.19	Succinic acid, pentadecyl 4-tert-butylphenyl ester	-	-
20.492	1.17	(R, R)-2-t-Butyl-5-methyl-1,3-dioxolan-4-one	$C_8H_{14}O_3$	104194-02-7
20.754	1.14	Eicosane	$C_{20}H_{42}$	112-95-8
20.823	1.11	2-Ethyl-5-n-propylphenol	$C_{11}H_{16}O$	72386-20-0
21.043	1.04	Dehydroacetic Acid	$C_8H_8O_4$	520-45-6
21.142	1.01	2,3-Dimethyl-undec-1-en-3-ol	$C_{13}H_{26}O$	-

21.339	0.98	Sorbic acid	$C_6H_8O_2$	110-44-1
21.512	0.94	2-Cyclohexen-1-one, 4-(3-hydroxy-1-butenyl)-3,5,5-trimethyl-	$C_{13}H_{20}O_2$	110114-85-7
21.919	0.94	Thunbergol	$C_{20}H_{34}O$	25269-17-4
22.1	0.89	Palmitic Acid	$C_{16}H_{32}O_2$	57-10-3
22.293	0.84	17-Octadecynoic acid, methyl ester	$C_{19}H_{34}O_2$	68950-90-3
22.728	0.8	3,9-Dimethyltricyclo [4.2.1.1(2,5)] decan-9-ol	$C_{12}H_{20}O$	-
22.81	0.75	D-Glucitol, 1,4-anhydro-	$C_6H_{12}O_5$	100402-56-0
22.923	0.75	Cyclopentanol, 3-methyl-, 1-acetate	$C_8H_{14}O_2$	24070-70-0
23.152	0.73	Eicosane	$C_{20}H_{42}$	-
23.297	0.68	Tridecane, 5-propyl-	$C_{16}H_{34}$	55045-11-9
23.559	0.68	Silane, trichlorooctadecyl-	$C_{18}H_{37}SiCl_3O_2Si$	71889-02-6
23.856	0.67	Ethanamine, N-ethyl-N-nitroso-	$C_4H_{10}N_2O$	55-18-5
23.941	0.66	Pyrene, 1-decylhexadecahydro-	$C_{26}H_{46}$	55191-41-8
24.062	0.64	Heptadecane, 3-methyl-	$C_{18}H_{38}$	383413-22-7
24.193	0.55	Furan, 2,3-dihydro-5-methyl-	C_5H_8O	1487-15-6
24.303	0.53	Silane, methylenebis[ethenyldimethyl-	$C_9H_{20}Si_2$	17865-60-0
24.482	0.5	5,7-Dimethyl-1,3-adamantanediol	$C_{12}H_{20}$	-
24.564	0.5	Hexadecyl nonyl ether	$C_{32}H_{66}O$	4113-12-6

Supplementary Figs

For "The endophytic strain Trichoderma asperellum 6S-2: An efficient biocontrol agent against apple replant disease in China and a potential plant-growth-promoting fungus"

Supplementary Figures:

Supplementary Figure S1. Solid fermentation process of *T. asperellum* 6S-2 in shallow plate. a: Fermentation substrate loading and fermentation start; b: *T. asperellum* (6S-2) hyphae formation; c: *T. asperellum* (6S-2) spore formation; d: Fermentation finish; e: Spore powder after crushing and sieving.

Supplementary Figure S2. Principal component analysis (PCA) of the T-RFLP profiles from healthy (H) and unhealthy (U) soils from MWW (a), MWL (b), MGD (c), QGP (d), QYC (e), YZY (f), YXX (g), LSW (h), and LSF (i). T-RFLP profiles were obtained following digestion of soil DNA using the Hha I enzyme. MWW: Wanggezhuang Village, Wanggezhuang Town, Muping District, Yantai City, Shandong Province; MWL: Luanjiatuan Village, Wanggezhuang Town, Muping District, Yantai City, Shandong Province; MGD: Dapankou Village, Guanshui Town, Muping District, Yantai City, Shandong Province; QGP: Panjialing Village, Guanli Town, Qixia City, Shandong Province; QYC: Changjiagou Village, Yangchu Town, Qixia City, Shandong Province; YZY: Yangjiazhuang Village, Zhongzhuang Town, Yiyuan County, Zibo City, Shandong Province; YXX: Xinzhuang Village, Xili Town, Yiyuan County, Zibo City, Shandong Province; LSW: Wantou Village, Shahe Town, Laizhou City, Shandong Province; LSF: Fengmaozhai, Shahe Town, Laizhou City, Shandong Province.

Supplementary Figure S3. Maximum-likelihood phylogenetic tree of the combined ITS and TEF genes from 44 taxa of *Trichoderma*. Sequences of *Protocrea pallida* and *Protocrea farinosa* were used as outgroups. Bootstrap values ≥70% and Bayesian posterior probabilities ≥0.90 are shown. The scale bar represents 10 substitutions per nucleotide position. The sequence from the fungal species obtained in this study is in bold and is 100% identical to that of the known species *Trichoderma asperellum* SZMC:24288.

Supplementary Figure S4. Effects of 6S-2 volatile substances and different concentrations of liquid fermentation extract on the growth radius of MR5 hyphae. (a) Hyphal radius with control or 6S-2 volatile substances in the two-part plate assay. $^{**}P < 0.001$ (n = 3, Student's *t*-test). (b) Hyphal radius with control or 6S-2 volatile substances in the sandwich plate assay. Ac, activated carbon. (c) Hyphal radius with different concentrations of 6S-2 fermentation extract. Bars with different letters are significantly different, $P < 0.05$ (n = 3; one-way ANOVA and Tukey's mean separation test).

Supplementary Figure S5. Mass spectrum of SPME-GC-MS

Supplementary Figure S6. Mass spectrum of HS-SPME-GC-MS

Supplementary Figure S7. The soil culturable microorganisms: bacteria (a); fungi (b); actinomycetes (c); and bacteria/fungi ratio (d); the real-time fluorescence quantification of four *Fusarium* species: *F. oxysporum* (e); *F. proliferatum* (f); *F. solani* (g); and *F. moniliforme* (h).

Supplementary Figure S1

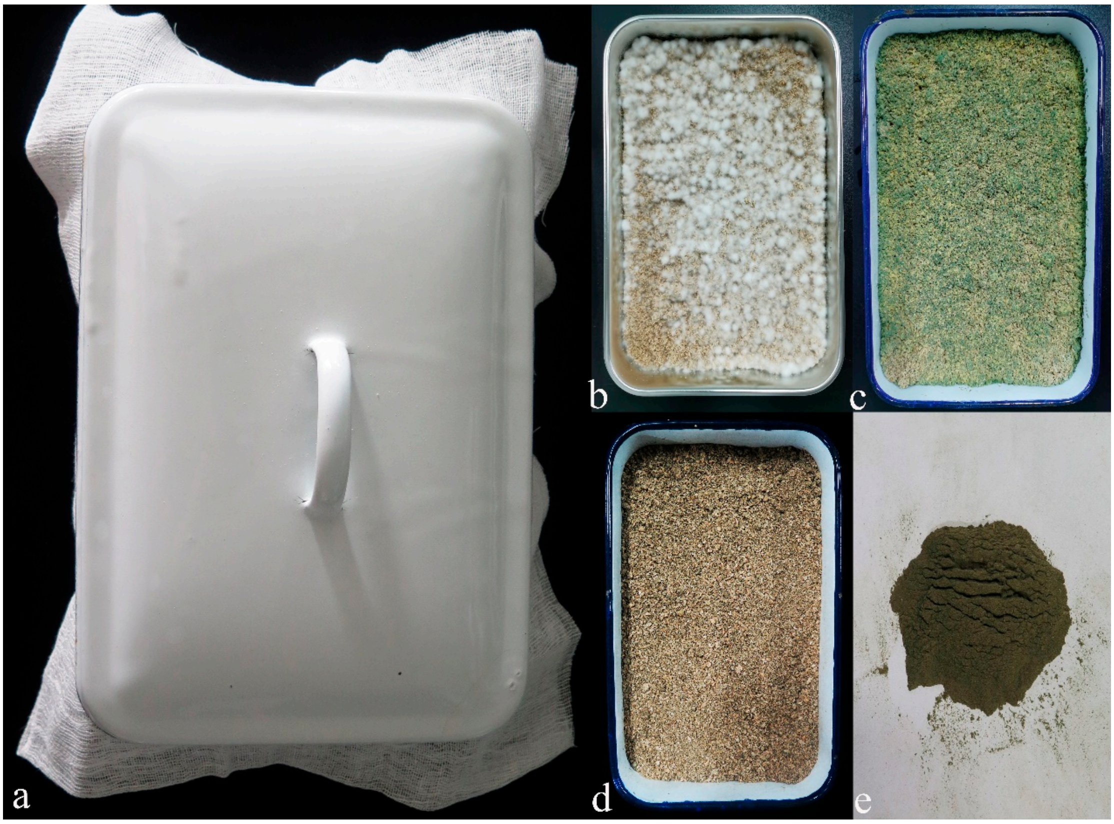

Supplementary Figure S2

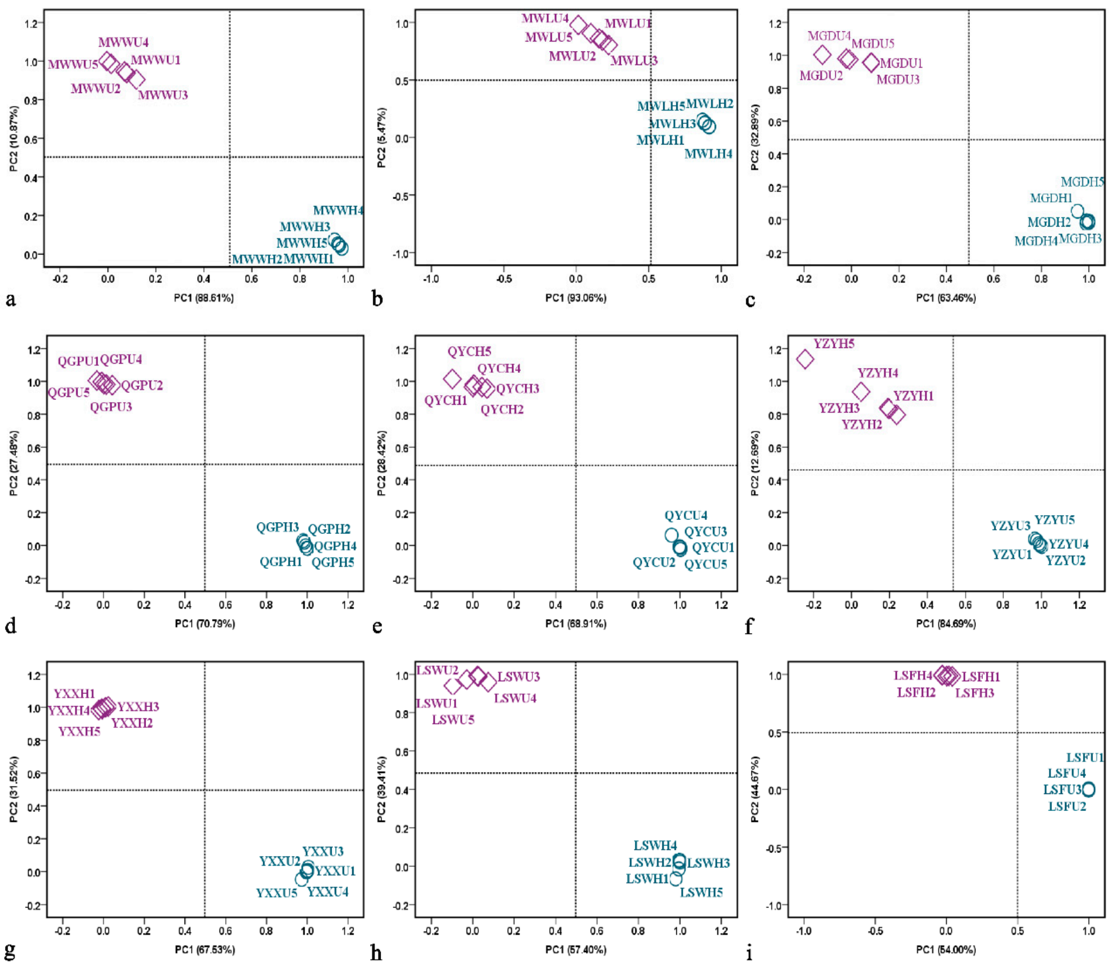

Supplementary Figure S3

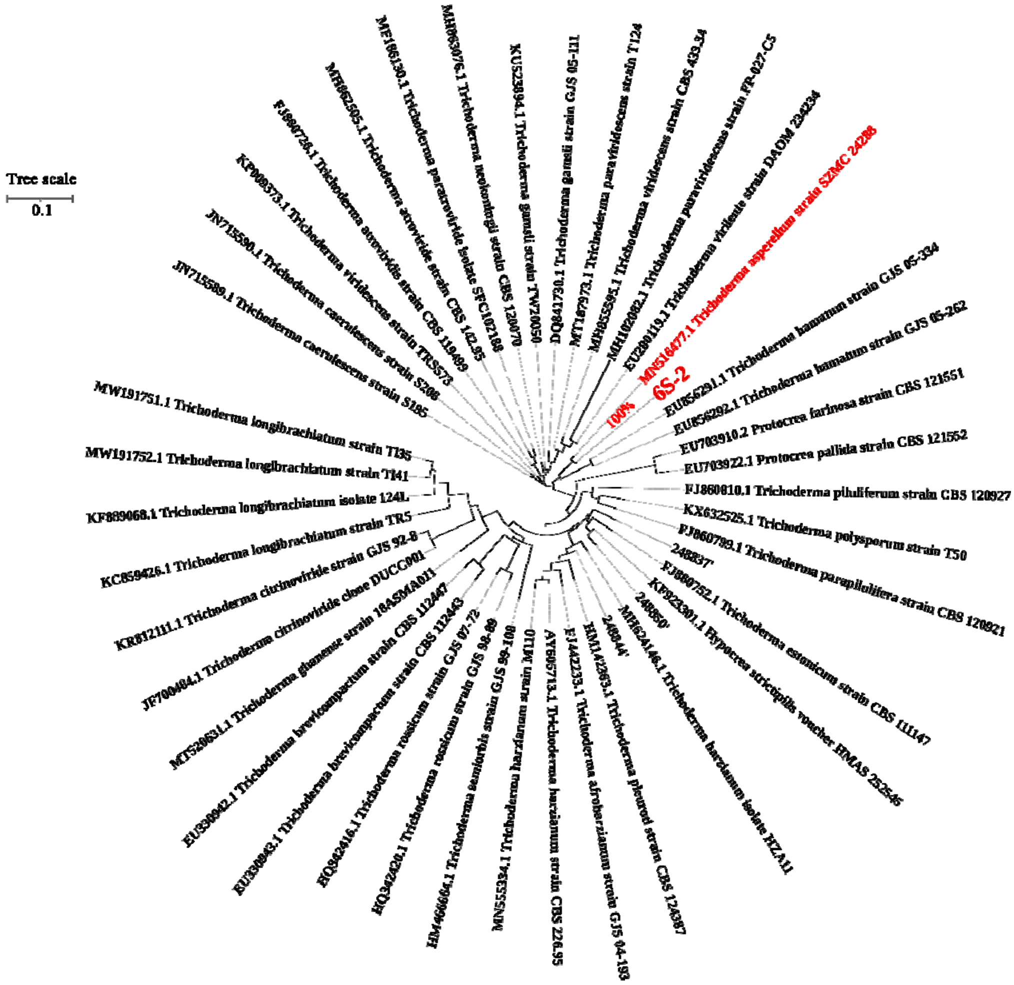

Supplementary Figure S4

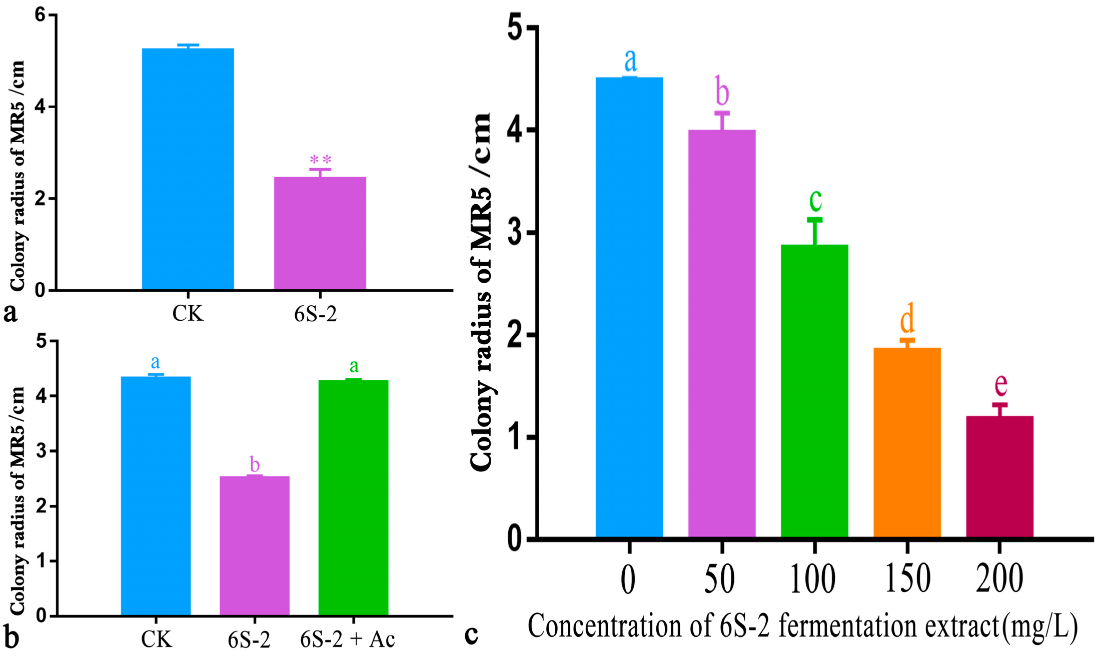

Supplementary Figure S5

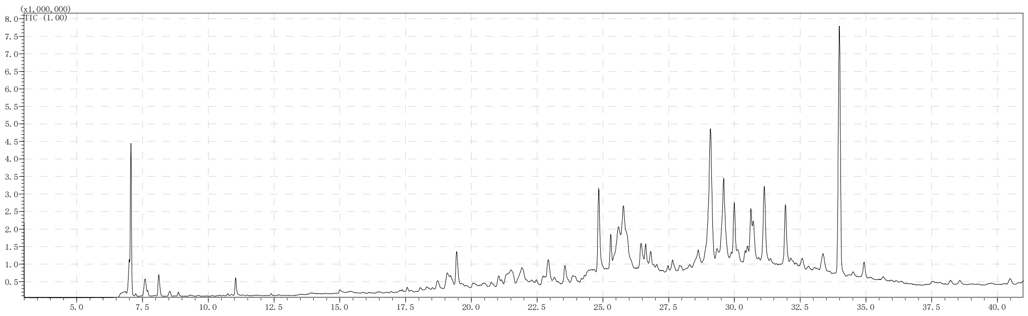

Supplementary Figure S6

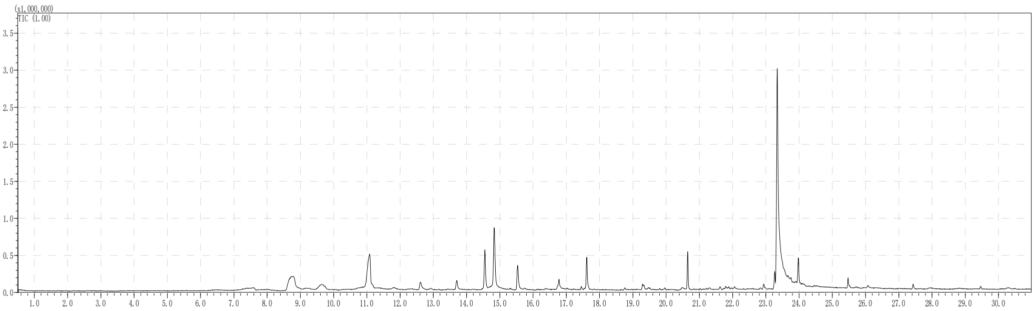

Supplementary Figure S7

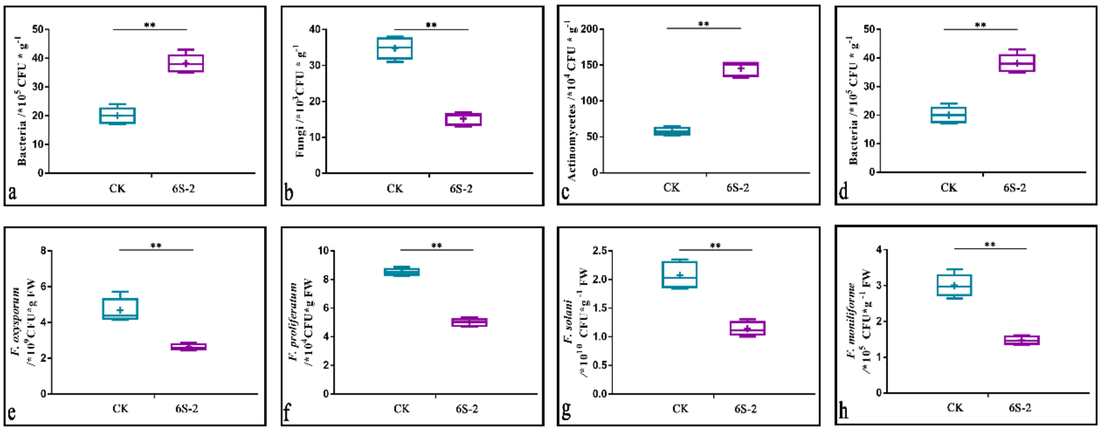

References

86. Mancini, V.; Murolo, S.; Romanazzi, G. Diagnostic methods for detecting fungal pathogens on vegetable seeds. *Plant Pathol.* **2016**, *65*, 691–703. https://doi.org/10.1111/ppa.12515.
87. Liu, Z.Y.; Zhang, W.B.; Tang, J.Y.; Shao, Y.H.; Dan, H.X. Difference analysis of biological characteristics of Valsa canker isolates in Jujube orchard. *North. Hortic.* **2018**, *11*, 49–54. https://doi.org/10.11937/bfyy.20173540. (In Chinese)
88. Zheng, H.; Qiao, M.; Lv, Y.; Du, X.; Zhang, K.-Q.; Yu, Z. New Species of *Trichoderma* Isolated as Endophytes and Saprobes from Southwest China. *J. Fungi* **2021**, *7*, 467. https://doi.org/10.3390/jof7060467.
89. Nautiyal, C.S. An efficient microbiological growth medium for screening phosphate solubilizing microorganisms. *FEMS Microbiol. Lett.* **1999**, *170*, 265–270. https://doi.org/10.1016/s0378-1097(98)00555-2.
90. Schwyn, B.; Neilands, J. Universal chemical assay for the detection and determination of siderophores. *Anal. Biochem.* **1987**, *160*, 47–56. https://doi.org/10.1016/0003-2697(87)90612-9.
91. Pérez-Miranda, S.; Cabirol, N.; George-Téllez, R.; Zamudio-Rivera, L.S.; Fernández, F. O-CAS, a fast and universal method for siderophore detection. *J. Microbiol. Methods* **2007**, *70*, 127–131. https://doi.org/10.1016/j.mimet.2007.03.023.
92. Dixit, R.; Singh, R.B.; Singh, H.B. Screening of antagonistic potential and plant growth promotion activities of *Trichoderma* spp. and fluorescent *Pseudomonas* spp. isolates against *Sclerotinia sclerotiorum* causing stem rot of French bean. *Legume Res. Int. J.* **2015**, *38*, 375–381. https://doi.org/10.5958/0976-0571.2015.00121.6.
